# Interrogation of the Protein-Protein Interactions between Human BRCA2 BRC Repeats and RAD51 Reveals Atomistic Determinants of Affinity

**DOI:** 10.1371/journal.pcbi.1002096

**Published:** 2011-07-14

**Authors:** Daniel J. Cole, Eeson Rajendra, Meredith Roberts-Thomson, Bryn Hardwick, Grahame J. McKenzie, Mike C. Payne, Ashok R. Venkitaraman, Chris-Kriton Skylaris

**Affiliations:** 1 Theory of Condensed Matter Group, Cavendish Laboratory, University of Cambridge, Cambridge, United Kingdom; 2 MRC Cancer Cell Unit Hutchison/MRC Research Centre, Cambridge, United Kingdom; 3 Cambridge Molecular Therapeutics Programme, Hutchison/MRC Research Centre, Cambridge, United Kingdom; 4 School of Chemistry, University of Southampton, Highfield, Southampton, United Kingdom; University of California, Davis, United States of America

## Abstract

The breast cancer suppressor BRCA2 controls the recombinase RAD51 in the reactions that mediate homologous DNA recombination, an essential cellular process required for the error-free repair of DNA double-stranded breaks. The primary mode of interaction between BRCA2 and RAD51 is through the BRC repeats, which are ∼35 residue peptide motifs that interact directly with RAD51 *in vitro*. Human BRCA2, like its mammalian orthologues, contains 8 BRC repeats whose sequence and spacing are evolutionarily conserved. Despite their sequence conservation, there is evidence that the different human BRC repeats have distinct capacities to bind RAD51. A previously published crystal structure reports the structural basis of the interaction between human BRC4 and the catalytic core domain of RAD51. However, no structural information is available regarding the binding of the remaining seven BRC repeats to RAD51, nor is it known why the BRC repeats show marked variation in binding affinity to RAD51 despite only subtle sequence variation. To address these issues, we have performed fluorescence polarisation assays to indirectly measure relative binding affinity, and applied computational simulations to interrogate the behaviour of the eight human BRC-RAD51 complexes, as well as a suite of BRC cancer-associated mutations. Our computational approaches encompass a range of techniques designed to link sequence variation with binding free energy. They include MM-PBSA and thermodynamic integration, which are based on classical force fields, and a recently developed approach to computing binding free energies from large-scale quantum mechanical first principles calculations with the linear-scaling density functional code onetep. Our findings not only reveal how sequence variation in the BRC repeats directly affects affinity with RAD51 and provide significant new insights into the control of RAD51 by human BRCA2, but also exemplify a palette of computational and experimental tools for the analysis of protein-protein interactions for chemical biology and molecular therapeutics.

## Author Summary

The atomic scale interactions that occur at the interfaces between proteins are fundamental to all biological processes. One such critical interface is formed between the proteins, human BRCA2 and RAD51. BRCA2 binds to and delivers RAD51 to sites of DNA damage, where RAD51 mediates the error-free repair of double-stranded DNA breaks. Mutations in BRCA2 have been linked to breast cancer predisposition. Therefore, an accurate picture of the interactions between these two proteins is of great importance. BRCA2 interacts with RAD51 via eight “BRC repeats” that are similar, but not identical, in sequence. Due to lack of experimental structural information regarding the binding of seven of the eight BRC repeats to RAD51, it is unknown how subtle sequence variations in the repeats translate to measurable variations in their binding affinity. We have used a range of computational methods, firstly based on classical force fields, and secondly based on first principles quantum mechanical techniques whose computational cost scales linearly with the number of atoms, allowing us to perform calculations on the entire protein complex. This is the first study comparing all eight BRC repeats at the atomic scale and our results provide critical insights into the control of RAD51 by human BRCA2.

## Introduction

The human breast cancer suppressor protein BRCA2 controls the functions of the RAD51 recombinase, an enzyme conserved in all kingdoms of life, which carries out the strand exchange reaction central to homologous DNA recombination (HDR) [1]. This essential cellular pathway is responsible for the error-free repair of DNA double strand breaks and is central to the maintenance of genome integrity and the prevention of diseases such as cancer [2].

Attempts to understand the role of BRCA2 in the regulation of HDR have been primarily driven by biochemical and cellular biological studies using regions of the full-length protein, amenable to cellular, biochemical and structural analyses. Two regions in the BRCA2 protein have been shown to interact directly with RAD51. The “BRC repeat” is a conserved motif of BRCA2 of approximately 35 amino acids that is thought to be the primary mode of interaction with RAD51. All known BRCA2 orthologues have been shown to contain at least one BRC repeat motif, but curiously the number of BRC repeats present varies between orthologues ranging from one (e.g. *Caernorhabditis elegans* Brc-2 and *Ustilago maydis* Brh2) to fifteen (e.g. *Trypanosoma brucei*) [3]. All vertebrate Brca2 proteins contain eight BRC repeats, clustered into a single large exon located in the central portion of the protein and show significant conservation of sequence and inter-repeat spacing [4]. The interaction between the BRC repeats of human BRCA2 and RAD51 has been characterised predominantly through structural and biochemical approaches and regulates many of RAD51's activities including RAD51 oligomerisation, and its ordered assembly on single-stranded or double-stranded DNA substrates to control the stepwise events of the strand exchange reaction [5], [6].

A second motif, unrelated in sequence to the BRC repeats, is found at the C-terminus of BRCA2 and, uniquely, is capable of interacting only with oligomerised RAD51 species in the presence or absence of DNA [7], [8]. A further major distinction is that this motif has no significant impact on the execution of HDR by RAD51, but rather links the disassembly of RAD51 complexes that form during HDR to the timing of entry into mitosis [9].

Three pieces of evidence suggest that the BRC repeats of human BRCA2 regulate RAD51-mediated strand exchange. Firstly, BRCA2-deficient cells are defective in HDR [10], [11]. Secondly, it has been shown that a region of BRCA2 comprising all eight human BRC repeats, or a subset of repeats fused to a DNA-binding domain, are capable of stimulating RAD51-mediated HDR and additionally, in the latter case, partially rescuing the HDR defect in BRCA2-deficient cells [5], [6], [12]–[14]. Thirdly, recent biochemical characterisation of the BRC repeats in isolation, as well as the intact human Brca2 protein, shows that they can stimulate RAD51 assembly on single-stranded DNA and inhibit its assembly on double-stranded DNA, hence promoting the stepwise DNA transactions required for strand exchange [5], [6], [15]–[17].

The crystal structure of the complex between the fourth human BRC repeat, BRC4, and the catalytic core domain of RAD51, conserved between all RAD51 orthologues (RecA in eubacteria and RadA in the archaea), has provided mechanistic insights into how BRC peptides can interact with RAD51 [18]. Interestingly, the BRC4 repeat binding to RAD51 was shown to antagonise RAD51 oligomerisation by directly binding to the oligomerisation surface of RAD51 found at the protomer∶protomer interface in oligomerised RAD51 assemblies. Intriguingly, this interaction uses precise molecular mimicry, rather than steric obstruction, to bind to RAD51 using an evolutionarily convergent amino acid sequence. BRC4 binds RAD51 using the motif 1524-FHTA-1527 ( *Homo sapiens* BRCA2 numbering) to establish contacts with RAD51 otherwise utilised by the sequence 86-FTTA-89 in the linker region of an adjacent RAD51 protomer.

A binding mode of BRC repeats antagonistic to RAD51 oligomerisation is not inconsistent with its stimulatory role in controlling RAD51. It has recently been reported that all BRC repeats may harbour a specific motif architecture that allows binding modes with RAD51 that may be permissive for RAD51 oligomerisation [19]. The identification and characterisation of two modules in the BRC repeats highlights an “FxxA” module that antagonises oligomerisation and an “LFDE” module (by BRC4 sequence nomenclature) that does not affect oligomerisation (and is likely to be permissive for oligomerisation), and complementary binding pockets in RAD51. These findings also suggest that binding modes at the BRC repeat-RAD51 interface are conserved across all known BRC repeats, permit differential regulation of RAD51 and are in essence a new example of hotspot-mediated protein-protein interaction. These tetrameric modules, and the corresponding pockets in RAD51, have been demonstrated to harbour the majority of binding capacity of an entire BRC repeat and their integrity is required for cellular viability through a critical mechanistic role in HDR.

Although these experimental studies focused upon BRC4, a known “strong binder” of RAD51, it was also shown that this conserved motif architecture was predicted to be partially intact even in the fifth BRC repeat, BRC5, a “weak binder” of RAD51, as an “LFDE”-like module was present. Indeed, this module was able to reconstitute RAD51 binding and regulation of RAD51 assembly of DNA when fused to a functional “FxxA” module, derived from BRC4.

Despite significant sequence similarity between the BRC repeats of BRCA2, several studies have reported that these motifs display varying affinities for RAD51 [20]–[22]. The functional relevance of having multiple repeats of varying affinities for RAD51 remains unclear, but may engender tighter regulation of RAD51 behaviour in the more complex genomic environment of higher organisms. Indeed, the finding that BRC repeats use two modules to mediate structural and functional associations with RAD51 and the observation that some repeats, such as BRC5, may contain just one of the modules, albeit of high affinity, speak to this idea.

In this study, we have combined experimental determination of the relative affinities of human BRC peptides for RAD51 with an array of computational simulations that address the atomistic determinants of the behaviour of BRC repeat binding to RAD51. We have used classical molecular dynamics (MD) simulations to explore the interface between RAD51 and the different BRC repeats and also their cancer-associated mutations at a critical interaction hotspot. From these simulation trajectories we have obtained the binding free energies of different BRC-RAD51 complexes using not only classical force fields, but also our newly developed QM-PBSA technique [23], which includes in the calculations the first principles quantum mechanical energies of the entire complexes. Furthermore, we have performed computational alanine scanning mutagenesis studies [24] on the repeats in order to pinpoint the energetic hotspots and quantify their strength in terms of the energetic contribution of each residue and used the more rigorous thermodynamic integration approach to verify critical findings. Our calculations confirm previously reported experimental binding behaviour and provide a rationale for observed differential affinities of BRC repeats for RAD51. Encompassing a range of accuracy and computational expense, these approaches to studying this promiscuous interface between RAD51 and, potentially, multiple peptides, provide fresh mechanistic insights into the regulation of RAD51 by multiple BRC repeats and serve as a template for the interrogation of protein-protein interactions of significant biological interest, often not amenable to direct experimental assessment.

## Results

### Human BRC repeats display varying capacities to disrupt the BRC4-RAD51 interaction

Several studies have previously reported the variation in binding affinities of human BRC repeats to RAD51 [20]–[22]. However, a quantitative comparison of these repeats has not been provided and indeed the majority of experimental insights are based upon BRC4, a stronger binder of RAD51 for which a high-resolution crystal structure exists of the complex. Attempts to purify a homogeneous preparation of RAD51 in a monomeric state amenable to biophysical studies of interaction with BRC peptides with a view to providing thermodynamic parameters have not been successful. In order to circumvent this technical challenge, we have developed a fluorescence polarisation (FP) assay that indirectly measures binding by determining the ability of BRC peptides to act as soluble inhibitors of the BRC4-RAD51 interaction in order to gauge the relative binding affinities of each of the repeats.

This assumes that all BRC peptides can bind to the same surface of RAD51 and are, in essence, competing for the interface on RAD51 pre-bound by BRC4. As all known BRC repeats share common sequence fingerprints that are matched by complementary sequence fingerprints in eukaryotic RAD51 orthologues in species with a BRCA2 orthologue [3], and this binding specificity has been confirmed experimentally, this assumption is likely to extend across all known BRC-RAD51 interactions.

RAD51 used for experimental determination of relative binding affinity was the full-length protein that maintains the capacity to oligomerise. However it should be noted that the structure of the BRC4-RAD51 complex is monomeric and comprises only the core catalytic domain, lacking the first 97 residues of RAD51 comprising the N-terminus and linker region [18]. The interactions of the BRC4 peptide with RAD51 extend along the length of the peptide, including the “LFDE”-module at its C-terminus in the partial context of an 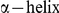. BRC binding to this region is likely to alter the N-terminal domain of RAD51 that is located in a conformation likely to sterically clash with the BRC peptide. The N-terminal domain is connected to the core catalytic domain through a flexible linker region and it is thought that this region of RAD51 engenders conformational flexibility in the N-terminus of RAD51 that is stimulated to accommodate, or be displaced by, BRC peptide binding. Indeed this conformational flexibility has been noted in several high resolution crystal structures of RAD51 orthologues and electron microscopic reconstructions of human RAD51 oligomeric assemblies on DNA in the presence of BRC peptides. The absence of the linker region in the construct used for crystallisation also renders the RAD51 species monomeric.

**Figure 1:**
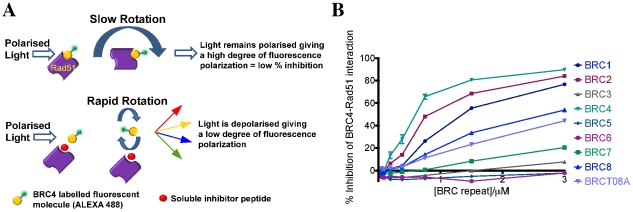
Relative binding affinities of BRC peptides for RAD51 via fluorescence polarisation assays. a) Principle behind the fluorescence polarisation assay. Alexa-labelled BRC4 peptide rotates slowly when in complex with RAD51 causing polarised light to remain polarised (top panel). Disruption of the BRC4-RAD51 complex by an unlabelled soluble competitor releases the Alexa-labelled BRC4 peptide, which now rotates rapidly causing depolarisation of incoming polarised light (bottom panel). b) Inhibition curves for all eight BRC repeats, as well as the BRC4 T1526A mutant. Full length wild type RAD51 protein was used at a concentration of 135 nM and Alexa488-BRC4 peptide at 10 nM. Peptides able to inhibit the BRC4-RAD51 interaction are detected by a reduction in fluorescence polarisation.

The outline of the FP assay for detection of disruption of the BRC4-RAD51 interaction is shown in Figure 1(a). Briefly, wild-type full length RAD51 was complexed with Alexa488-conjugated BRC4 and incubated with varying concentrations of each of the eight BRC repeats (unconjugated), present as unlabelled soluble competitive peptides.

In accord with the findings of several qualitative analyses [21], [22], the BRC repeats showed a well-defined relative order of competitive inhibition of the BRC4-RAD51 interaction (Figure 1(b)). BRC4 was the most potent competitive inhibitor, followed by BRC2 and BRC1. BRC8 showed a markedly weaker competitive inhibition. BRC7 and BRC3 showed mild competitive behaviour but failed to achieve 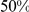 inhibition even at the highest concentrations of peptide (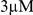) and BRC5 and BRC6, in accord with previous reports, showed no significant competition of the BRC4-RAD51 interaction. The BRC4 T1526A mutant (a previously reported non-binding mutant identified by sequential mutagenesis) [25] showed weak competitive inhibition relative to wildtype BRC4.

### Computational alanine scanning identifies two binding hotspots in BRC4

Understanding protein-protein interactions using computational methods is a major goal at the nexus between structural biology, biophysics and computational chemistry, but is often compromised by limitations of accuracy, high computational cost and the inability to simulate large systems. In this study, we combine a variety of computational methods, with a range of accuracy and computational expense, that are able to measure and rationalise protein behaviour in the context of existing macromolecular complexes. Such methods can help us achieve an understanding of a wide variety of problems relevant to the basic biology of all cellular processes reliant on protein-protein interactions to allow, for example, small molecule chemical intervention with therapeutic or chemical biological rationale.

We begin our analysis with a computational alanine-scanning mutagenesis study [24], [26] of BRC4 using the MM-PBSA method [27], [28]. This approach estimates the contribution of each residue to the free energy of binding at a protein-protein interface by mutating each residue in turn to alanine and measuring the effect of the mutation on the overall free energy of binding. This is done while accounting for the dynamical nature of the interactions and the effects of solvation. Such simulations are directly analogous to the experimental technique of alanine scanning mutagenesis [29], [30], which is used to identify “energetic hotspots” on protein-protein interfaces [31], [32].

**Figure 2:**
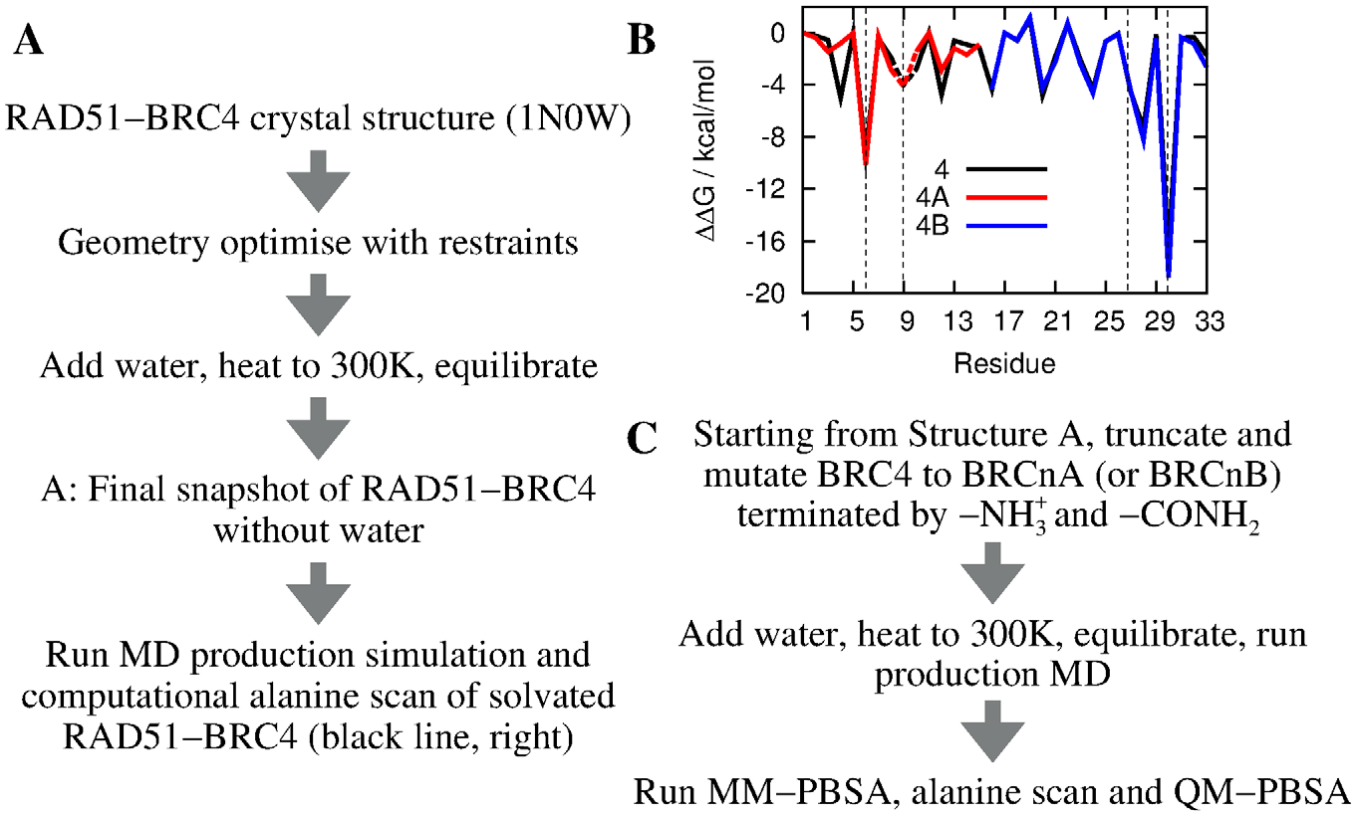
Computational alanine scanning mutagenesis identifies two binding hotspots in BRC4. a) Procedure for the MD simulations starting from the RAD51-BRC4 X-ray crystal structure. b) Computational alanine mutagenesis scan of the RAD51-BRC4 complex. Dashed lines indicate an alanine to glycine mutation. The two interaction hotspots (“FHTA” and “LFDE”) are denoted by vertical dashed lines and contribute significantly to the total free energy of binding. Alanine scans from separate RAD51-BRC4A and RAD51-BRC4B complexes are also shown and reproduce the behaviour of the full-length peptide (see next section). c) Simulation outline describing the generation of the eight RAD51-BRCnA structures from a snapshot of the RAD51-BRC4 MD simulation (Structure A).

Figure 2(a) summarises the MD procedure and Figure 2(b, black line) reports the change in binding free energy (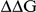) resulting from the mutation of the side chain of each residue of BRC4. As previously reported by Rajendra and Venkitaraman [19], this computational mutagenesis approach highlighted both F1524 and L1545/F1546/E1548 via alanine scanning and A1527 via glycine scanning as residues contributing significantly to the binding of BRC4 to RAD51. Thus, these results are both predictive and fully supportive of a model whereby two modules in the BRC repeats are involved in hotspot-mediated interaction with RAD51.

### MM-PBSA simulations are able to rank the affinity of the BRC peptides for RAD51 relative to RAD51-RAD51 self-oligomerisation

Having established that computational alanine scanning mutagenesis confirms the presence of two modules within BRC4 previously identified experimentally [19] that contribute to its interaction with RAD51, we sought to understand if further analysis could provide insights into the behaviour of the larger regions of the BRC peptides to establish why they displayed different experimental affinities for RAD51. As no high resolution structural information is available for human BRC repeats 1–3 and 5–8, and accurate biophysical interrogation is hindered by technical challenges in the purification of a suitable N-terminally-truncated monomeric RAD51 species, we turned to computational simulations to analyse the interactions of each of the BRC peptides with RAD51.

**Figure 3:**
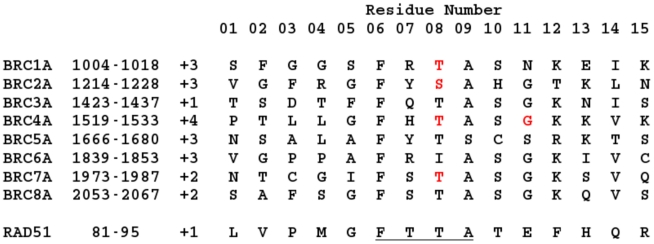
Sequence alignment of the 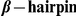 loop regions. Shown are the eight human BRC repeats used in the computational simulations, as well as the 15 residues of the ligand at the RAD51-RAD51 self-oligomerisation interface. BRCA2 residue numbering is shown in column two, but for simplicity, residues will be labelled 01-15 throughout this paper. The FxxA hotspot is underlined, net charges of each ligand are shown in column three as multiples of the electronic charge and sites of mutations studied here are highlighted in red.

In order to approach this problem, we used classical MD simulations of the N-terminal 15 residues of the BRC peptides, denoted “BRCnA” with residue sequences shown in Figure 3. We chose this region for two key reasons. Firstly, given that the full length RAD51 is used for FP assays and only the core catalytic domain is used in simulations, a simulation including the C-terminal 18 residues of BRC peptides (“BRCnB”) would be questionable as this region may be sterically interdependent with the N-terminal domain of RAD51, which is missing in the simulated complex. Attempts to simulate the BRC5B peptide region suggested much weaker binding than observed experimentally and binding modes that did not conform to the crystallised RAD51-BRC4 complex (Figure S1). Secondly, the BRCnA region contains the FxxA module that has a defined functional effect (antagonism of RAD51 oligomerisation) that could be later benchmarked against the binding energy between RAD51 protomers in an oligomeric assembly. Care should be taken when comparing the results of MM-PBSA simulations and our FP assays, as any contribution to binding affinity caused by sequence variation outside the truncated BRCnA peptides is neglected in our computational model. However, Figure 2(b) confirms that use of separate RAD51-BRC4A and RAD51-BRC4B trajectories gives very similar binding behaviour to the full RAD51-BRC4 complex around the significant hotspot regions, indicating that our conclusions concerning the effects of sequence variation in the BRCnA half-peptides are unaffected by our choice of truncation of the experimental peptide. The tail regions of the BRC4A peptide show more variation in the alanine scan of Figure 2(b) since they are more mobile than the residues located in the hotspot. As such, they may become trapped in local minima of the free energy landscape for the duration of the simulation, artificially affecting the free energy of binding calculated by MM-PBSA. With this in mind, throughout this study, we used a combination of MM-PBSA to obtain the total free energy of binding and computational alanine scanning to quantify the contribution of each of the BRCnA peptides in the significant hotspot region, and thus discern the effects of sequence variation.

We used the MD protocol outlined in Figure 2(c) to generate structures of the RAD51-BRCnA interfaces starting from the RAD51-BRC4 crystal structure. BRCnA peptides derived from BRC repeats 1–3 and 5–8 were generated by mutating selected side chains of the BRC4A structure as described in the methods. Here, we assume that each BRC repeat folds in the same manner as BRC4A since no changes in secondary structure are expected to occur on the time scales of these simulations, although significant, localised variations in binding behaviour were observed for some BRC repeats.

**Figure 4:**
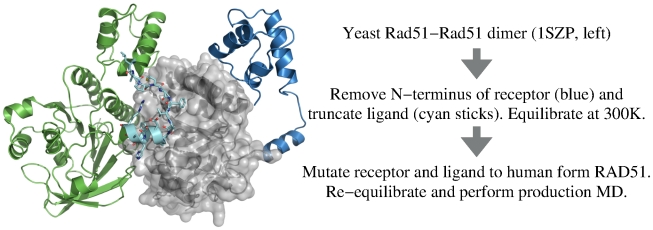
Outline of the simulation of the humanised RAD51-RAD51 oligomeric assembly. The starting structure is that of the *Saccharomyces cerevisiae* dimer. The final complex consists of the core catalytic domain of human form RAD51 receptor (silver) and a 15 residue RAD51 “ligand” (cyan).

Given the importance of the BRCnA repeats in antagonism of RAD51-RAD51 oligomerisation, the relative binding free energies at this protein-protein interface is of significant mechanistic interest. Despite sequence similarity in the hotspot region itself (FHTA in BRC4A mimics FTTA in RAD51), the protein sequence used by RAD51 to self oligomerise is known to partially comprise a helical region [33], which is unlikely to form spontaneously from the 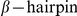 structure of BRC4 over the time scale of these simulations. Figure 4 summarises the alternative method used here to generate the RAD51-RAD51 interface starting from a dimeric unit from the crystal structure of the *Saccharomyces cerevisiae* Rad51 (see methods) and retaining 15 residues of the Rad51 ligand, to match the sequence register of the 15 residues of the BRCnA repeats.

**Figure 5:**
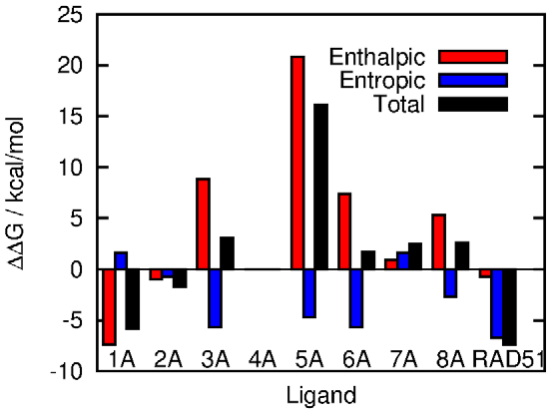
Binding free energy of each of the eight BRC repeats and the RAD51-RAD51 complex. Binding free energies are relative to RAD51-BRC4A and are broken down into enthalpic, which is a sum of the gas phase binding energy and free energy of solvation, and entropic, which is due to changes in solute degrees of freedom upon binding and is calculated by normal modes analysis.

We have used the single trajectory classical MM-PBSA technique, with the gas phase binding entropy of the molecules calculated using a normal modes analysis, to compute the relative free energies of binding of each of the eight BRCnA repeats to RAD51, and compared them to the binding free energy of the RAD51-RAD51 interface. With the exception of BRC5A, Figure 5 shows that the relative free energies of binding of the BRC repeats to RAD51 are very similar, which reaffirms the requirement for very precise measurements of their affinities. Interestingly, MM-PBSA predicts the truncated RAD51 ligand to be the strongest binder to the RAD51 oligomerisation interface, although the difference is mostly entropic and this term is usually assumed to carry the greater uncertainty. Three of the BRC repeats (BRC1A, BRC2A and BRC4A, in that order) bind with affinity comparable to RAD51. Our combination of FP assays and MM-PBSA indicates that BRCA2 also contains five more weakly-bound BRC repeats and the sequence differences that give rise to this variation in affinity will be investigated in the following sections.

### QM-PBSA analysis confirms the relative binding free energies of BRC repeats

The relative binding free energies of the BRCnA repeats to RAD51 as determined by MM-PBSA (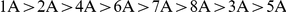) are in reasonable qualitative agreement with the inhibition order of the BRC repeats derived from FP assays (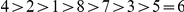). Notable discrepancies are the over-estimation of the binding affinity of both BRC1A and BRC6A in the MM-PBSA approach. One reason for this may be limitations of the computational system, such as the neglect of the 18 BRCnB C-terminal residues and the N-terminal domain of RAD51. Another reason may be limitations of the force field used to describe the interactions between receptor and ligand, which on this length scale are inherently quantum mechanical in nature.

To address the limitations in accuracy of classical force fields, caused by their dependence on a large number of parameters and their inherent inability to describe charge transfer and polarisation we have recently developed a new computational approach that allows us to calculate, from first principles quantum mechanics (QM), the binding free energy of biomolecular complexes consisting of thousands of atoms [23]. In this QM-PBSA approach binding energies are obtained with Density Functional Theory (DFT) calculations which do include charge transfer and polarisation effects. Here, we use QM-PBSA calculations to re-assess the free energy of binding of four of the studied complexes, RAD51-BRC4A (reported in a previous work [23]), RAD51-RAD51, and the two discrepancies between experiment and MM-PBSA, RAD51-BRC1A and RAD51-BRC6A.

**Figure 6:**
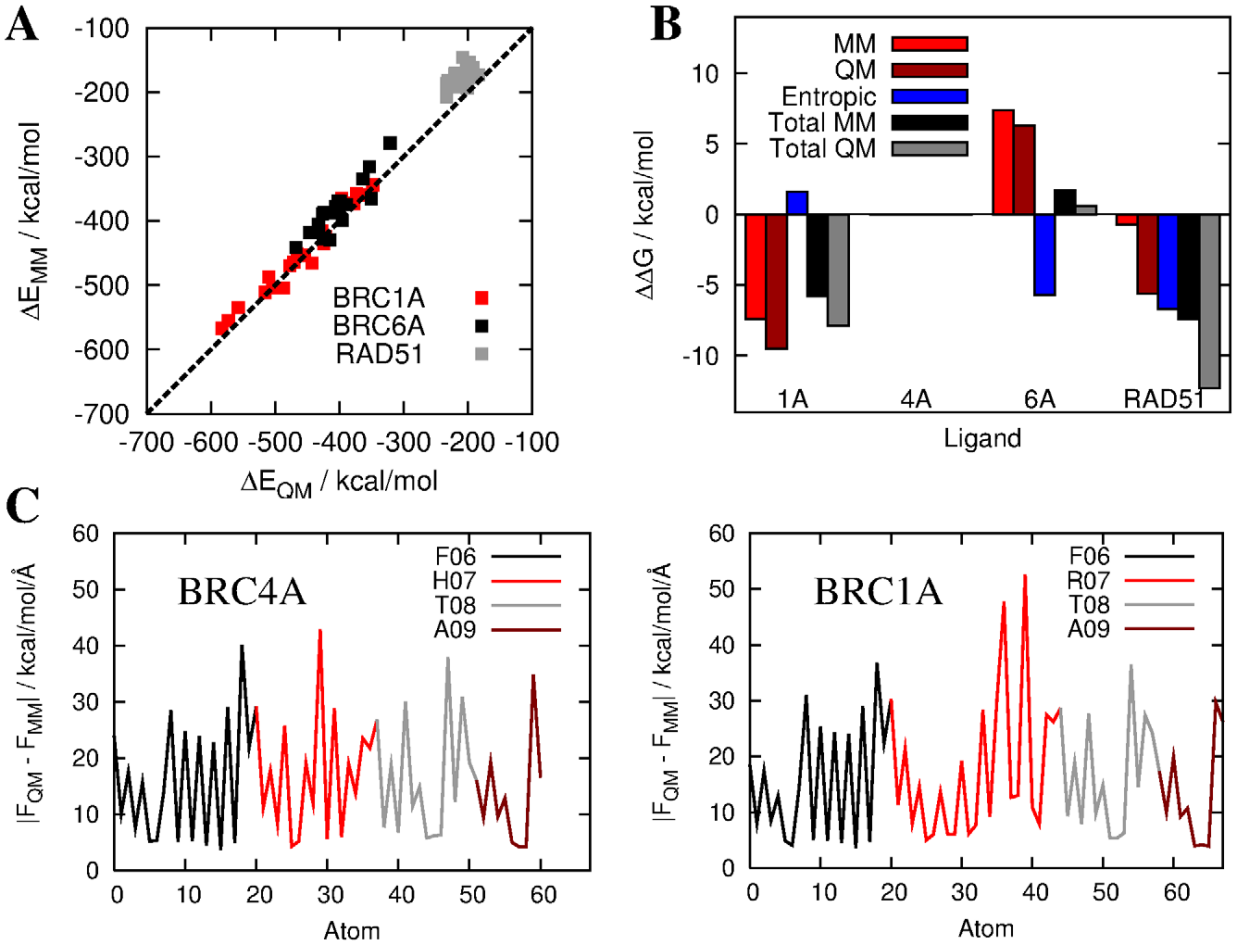
Results of QM-PBSA analysis of the RAD51-BRCnA complexes. a) Correlation between the QM and MM total gas phase binding energies for the complexes between RAD51 and BRC1A, BRC6A and RAD51. b) Contributions to the free energy of binding (relative to RAD51-BRC4A) in MM-PBSA and QM-PBSA. Similar relative binding affinities are observed in MM-PBSA and QM-PBSA. c) Magnitude of the vector force errors on atoms of the ligand in the “FxxA” hotspot region from simulations of the RAD51-BRC4A and RAD51-BRC1A complexes.

Figure 6(a) reveals that there is very good correlation between the gas phase binding energies calculated within MM-PBSA and QM-PBSA. The classical force field is very accurate for the RAD51-BRC1A interaction but under-estimates both the RAD51-BRC6A and RAD51-RAD51 gas phase binding energies. The relative free energies of binding are calculated within QM-PBSA by combining these gas phase binding energies with the scaled solvation free energy and the classical relative entropy change of the solutes upon binding. Figure 6(b) reveals that the binding order of the investigated complexes is the same as predicted by MM-PBSA (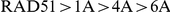), although the relative binding free energy of RAD51-BRC1A and RAD51-RAD51 are under-estimated by 2–5 kcal/mol in MM-PBSA.

Despite significantly improving the calculation of the gas phase quantity in the MM-PBSA scheme, the QM-PBSA method is still potentially subject to inaccuracies. Firstly, the error in the entropy contribution, calculated by classical normal modes analysis, may be large and future improvements in this area should concentrate on increasing the precision of this term. Secondly, the binding energy is calculated by sampling snapshots taken from the classical MD trajectory, which assumes adequate sampling of the ligand's conformational space by the classical force field. To demonstrate this latter limitation, in Figure 6(c), the magnitude of the vector difference between the QM and MM forces averaged over the snapshots is plotted for each ligand atom in the hotspot region of the RAD51-BRC4A and RAD51-BRC1A complexes. The differences are generally small indicating that the QM configurational space is well sampled by the classical force field. However some discrepancies exist in polar groups, especially the R07 side chain in BRC1A, and methods to force-match the force field to the QM forces based on the local environment of the proteins [34], [35] are the subject of ongoing work.

### Atomistic determinants of BRC repeat affinity are revealed by computational simulations of RAD51-BRCnA complexes

Having established, via three complementary methods, that the BRC repeats show a defined relative order of affinity for RAD51, and in concert with the identification of a sequence motif architecture comprising two specific interaction modules across all BRC repeats with complementary binding energy hotspots in RAD51, we sought to understand why each of the different BRC repeats varied in their affinity to RAD51.

**Figure 7:**
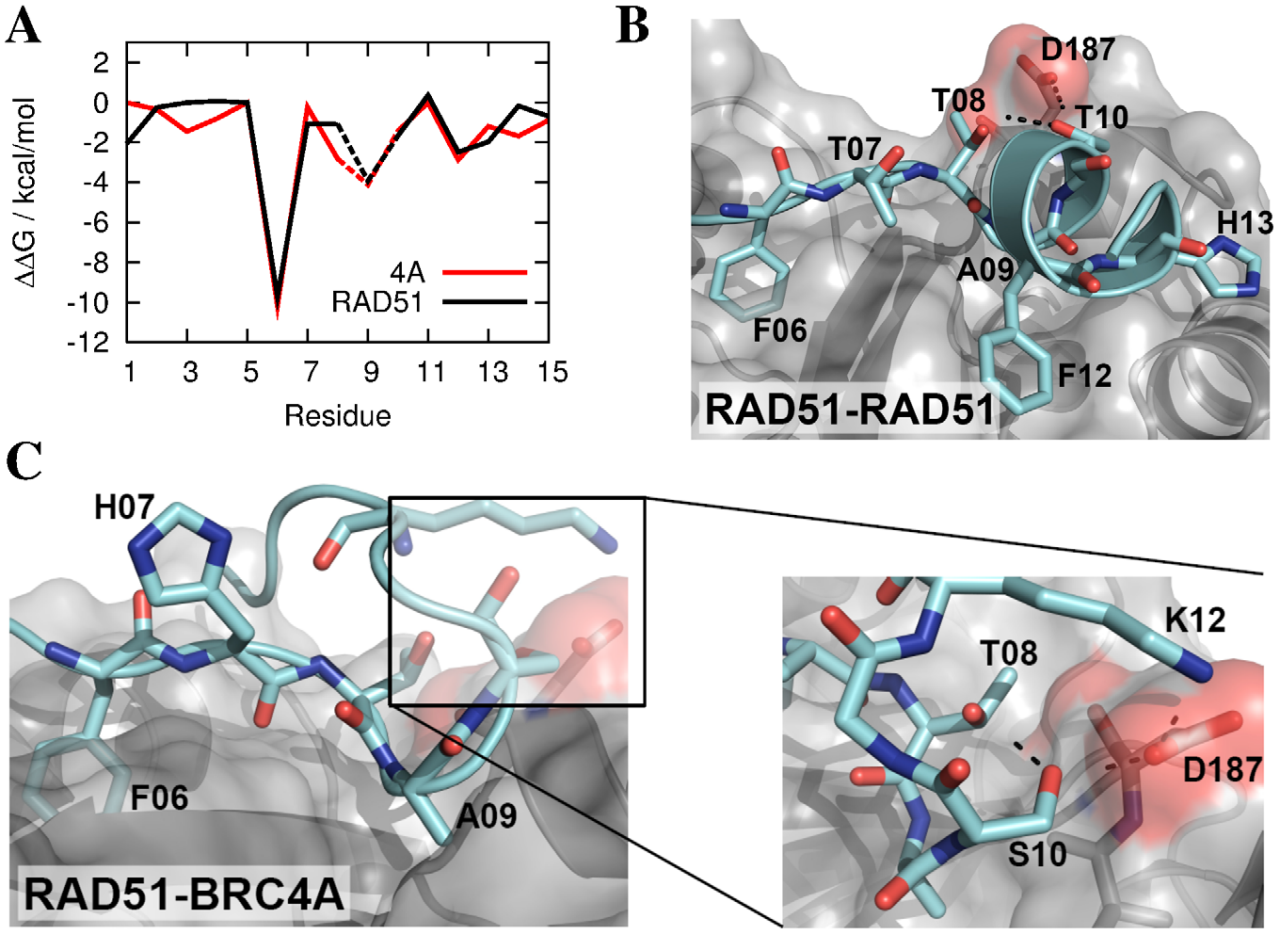
MD simulations of the RAD51-RAD51 and RAD51-BRC4A complexes. a) Computational alanine scan, comparing contributions of each residue to binding, reveals a similar interaction pattern in the RAD51-RAD51 dimer to that of its antagonist, BRC4A. Dashed lines indicate an alanine to glycine mutation. b) Snapshot of the RAD51-RAD51 complex in the region of the interaction hotspot. The RAD51 receptor is shown in silver and significant hydrogen bonds as dashed lines. c) Snapshot of the RAD51-BRC4A interaction from MD simulation and close up of the four-way interaction between T08, S10, K12 of BRC4A and D187 of RAD51.

We first utilised computational alanine-scanning mutagenesis, which we have already shown to be predictive of the hotspot-mediated interaction of BRC4 with RAD51, to probe the RAD51-RAD51 and RAD51-BRC4A interaction interfaces for differences in binding free energy that could be associated with sequence variation. The RAD51-RAD51 oligomeric interface differs from the RAD51-BRC4A interface in both structure (the hairpin is replaced by a helical segment) and interaction type (dispersion interactions account for approximately 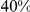 of the QM gas phase binding energy, compared to just 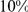 in BRC4A). Yet the binding free energies and even each residue's individual contribution to binding, revealed by the computational alanine scan, are remarkably similar (Figure 7(a)).

A representative snapshot of the RAD51-RAD51 complex is shown in Figure 7(b). The interactions of the FxxA hotspot motif, namely F06 and A09 hydrophobic interactions and the backbone inter-protein hydrogen bonds of residue T07 (which do not contribute to the alanine scan), are present in RAD51 as well as BRC4. In the RAD51-RAD51 complex, the alanine scan reveals that residues T10 and F12 provide significant additional contributions to binding. T10 forms an intermittent hydrogen bond with RAD51 via D187 with an occupancy of 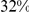. F12 forms contacts with residues F166, P168 and Y191 of RAD51. Hydrophobic contact is also formed to some extent between H13 and the RAD51 surface.

The major differences between the RAD51-RAD51 self-oligomerisation interface and the RAD51-BRC4A complex are the increased contribution to binding of residue T08 in the latter and the removal of the hydrophobic F12, which is replaced by the charged residue K12 with little change in binding affinity. In order to bind to the RAD51 interface, K12 forms a salt bridge with D187 of RAD51 (Figure 7(c)) and, to accommodate this change in interaction, BRC4 adopts a 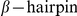 structure, whose stability was noted in a previous study [36]. In fact, the backbone interactions between residue 08 and residues 11 and 12 that span the hairpin are found here in all eight simulations. Residue T08 appears to contribute further to hairpin stability by forming side chain hydrogen bonds with the side chain of S10 and the backbone of K12 (Figure S2(a)) and hydrophobic contacts with the methylenes of the K12 side chain and the D187 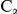 atom (Figure S2(b)). The latter interaction accounts for the higher contribution to binding of T08 in RAD51-BRC4A relative to RAD51-RAD51 and by interacting simultaneously with residues S10, K12 and D187 (Figure 7(c)), we speculate that T08 stabilises the hairpin interaction with RAD51. This hypothesis is supported by the close homology between five of the eight repeats (BRC1A, BRC3A, BRC4A, BRC7A and BRC8A) in the hotspot region, all of which contain the sequence –FxTASxK– and have very similar alanine mutagenesis scans to RAD51-BRC4A (Figure S3).

The information gained from alanine mutagenesis can be used to resolve the discrepancy between MM-PBSA (or QM-PBSA) and our FP assays. The relative free energies of binding of these five BRC4A-like repeats, appear to be determined not by any sequence variation in the hotspot region, but by the strength of the electrostatic attraction between the ligands of varying net positive charge (Figure 3) and the negatively charged receptor. If we rank these repeats in order of increasing charge (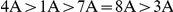), we observe very good agreement with the relative abilities of these repeats to compete the RAD51-BRC4 interaction in FP assays (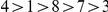). The unexpectedly strong affinity of BRC1A for RAD51 in MM-PBSA appears to be caused by its strongly bound C-terminus (Figure S3), which, as in the 1N0W RAD51-BRC4 crystal structure, is expected to point away from RAD51 in the context of the full BRC1 peptide, and is therefore an artefact of our computational model.

### Alanine to serine mutation in BRC5 reduces free energy of binding to RAD51

**Figure 8:**
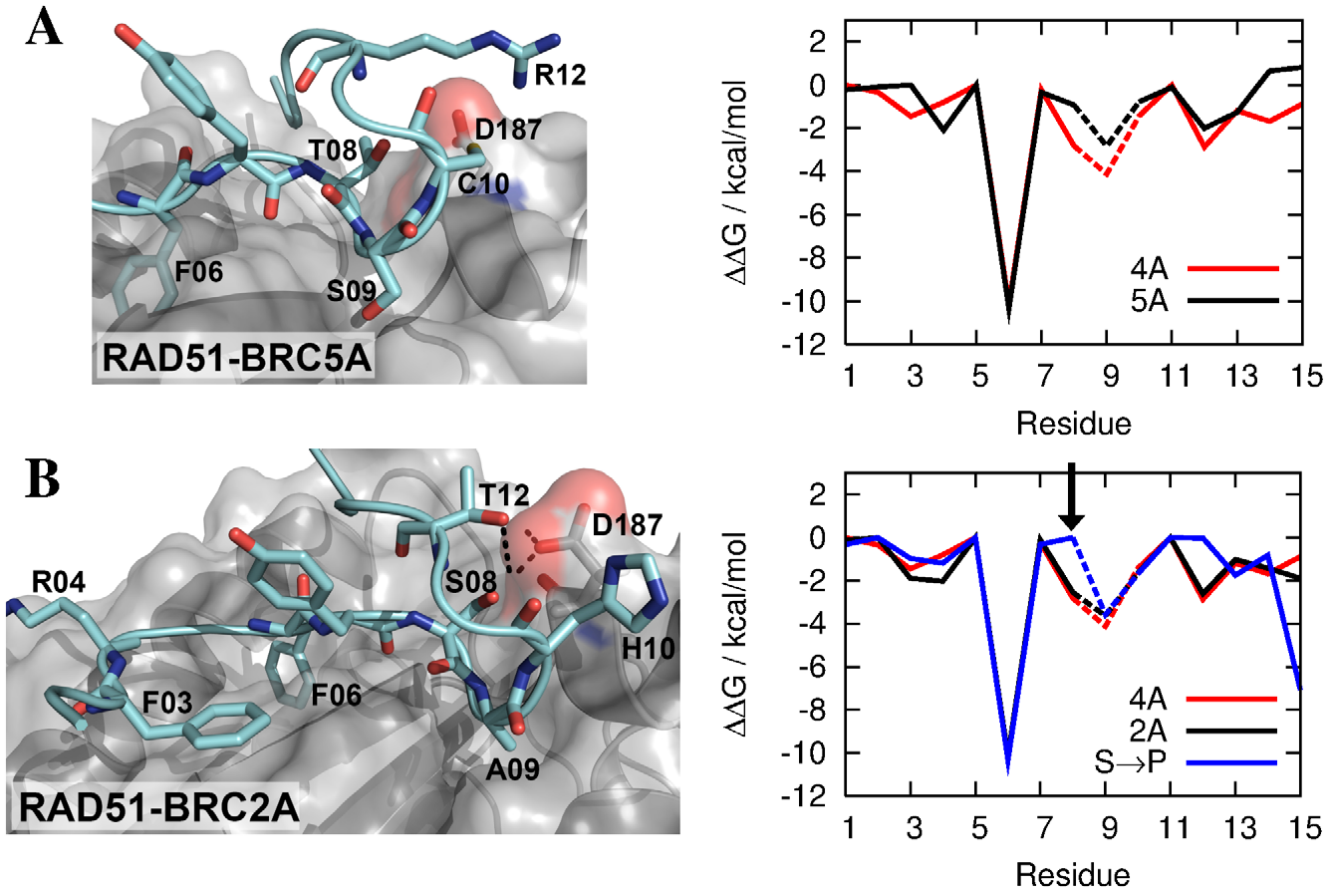
MD simulations of the RAD51-BRC5A and RAD51-BRC2A complexes. Snapshots of a) the RAD51-BRC5A and b) the RAD51-BRC2A protein-protein interfaces from MD simulations and corresponding computational alanine mutagenesis scans. Dashed lines indicate an alanine to glycine mutation. BRC5A is noticeably less strongly bound than BRC4A in the hotspot region, particularly at residue 09 where alanine is substituted by serine. The BRC2A alanine scan is very similar to that of BRC4A despite the differences in adopted binding modes. The arrow indicates the position of the S08P mutation in BRC2A, which reduces contributions to binding from residues 08 and 12 in the mutated complex.

As we have shown in the previous section, five of the eight BRC repeats use very similar motifs to bind RAD51. BRC5, however, replaces the sequence –FxTASxK– with –FxTSCxR–, the most notable change in sequence being the replacement of A09 in a hydrophobic pocket in RAD51 by the polar residue S09. Isothermal titration calorimetry measurements have recently shown that a single A09S mutation in BRC4 is sufficient to significantly reduce the rate constant for the RAD51-BRC4 association reaction and, in turn, reduce the capacity of BRC4 to dissociate the RAD51-DNA complex [37]. In Figure 8(a), we compare a computational alanine scan of the RAD51-BRC5A interface with that of the RAD51-BRC4A interface. The most interesting difference between the two curves is at position 09. Although S09 remains bound throughout the MD simulation, its contribution to the binding free energy is 1.3 kcal/mol lower than the A09 contribution in RAD51-BRC4A. This energy difference is sufficient to explain experimental observations of loss of binding affinity upon A09S mutation in BRC4 [37] and may be rationalised by considering the relative solvation free energies of the two residues, which are accounted for naturally in the MM-PBSA scheme.

The binding mechanism of BRC5A to RAD51 is otherwise very similar to the RAD51-BRC4A complex (Figure 8(a)). Cysteine is less polar than serine, which it replaces as residue 10, and forms neither intra-protein hydrogen bonds with T08 nor inter-protein hydrogen bonds with D187. The R12-D187 interaction is present, though is weaker than the K12-D187 interaction that it replaces in the BRC4A-like repeats. Overall, both FP assays and MM-PBSA predict a very weak interaction between RAD51 and BRC5.

### MD simulations of RAD51-BRC2A reveal alternative binding modes for BRC repeats containing significant sequence differences

The use of computational simulation, in particular MD, allows the investigation of dynamical motion and access to structures that are not amenable to experimental structural determination. This is particularly relevant for the BRC repeat, BRC2A, which differs significantly in sequence from BRC4A in the hotspot region, replacing the sequence –FxTASxK– with –FxSAHxT–. Analysis of the RAD51-BRC2A MD trajectory reveals that H10 does not form hydrogen bonds within the BRC2A hairpin or directly with RAD51, which is the role of S10 in BRC4A. Yet BRC2A is among the most strongly bound repeats according to both FP assays and MM-PBSA.

By using MD simulations to explore the conformational space of the receptor-bound ligand and alanine scans to probe the contribution of each residue to the binding free energy, we are able to rationalise the high affinity of BRC2A for the RAD51 interface. Figure 8(b) reveals a different binding mode to that observed in the crystal structure of the RAD51-BRC4 complex. Firstly, the computational alanine scan reveals contributions to binding from residues F03, which forms a hydrophobic contact with the RAD51 surface, and R04, which is due to longer-ranged electrostatic effects. Secondly, the S10-D187 hydrogen bond is replaced by S08-D187 and the T12-D187 bond is present for a higher proportion of the simulation than the K12-D187 interaction in, for example, RAD51-BRC4A (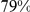 vs. 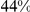). This change in binding pattern appears to introduce strain into the RAD51-BRC2A hotspot interface. The two hydrogen bonds formed between the backbone of residue 07 and RAD51 are well conserved in the other seven repeats, but here are 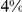 and 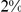 longer than at the RAD51-BRC4A interface (Figure S4). Despite a significant variation in residue sequence in the BRC2A hotspot region compared to the other BRC repeats, the similarity of its binding free energy and alanine scan with those of BRC4A is striking.

Armed with this knowledge, we can propose mechanisms for binding of the different BRC repeats to RAD51 with varying affinity, with implications for the regulation of HDR. BRCA2 is mutated in a significant proportion of individuals with familial breast and ovarian cancer [38], [39]. However, of the many sequence alterations in BRCA2 that have been found in cancer samples (Breast Cancer Information Core, http://research.nhgri.nih.gov/bic/) [18], it remains unclear which represent silent genetic variations and which represent pathogenic mutations. This remains a major problem in the field. We have therefore sought to test whether our atomistic simulations of BRCnA-RAD51 complexes might reveal information concerning the ability of cancer-associated BRCA2 alterations to affect the interaction between BRCA2 and RAD51. With this in mind, we have performed a series of additional simulations on carefully selected single residue mutations, which are designed both to test our predictions regarding the amino acid sequences that give rise to differential binding affinities in the BRC repeats and to examine the effects on binding of single residue mutations associated with cancer development.

One such potentially pathogenic mutation is the S08P substitution in BRC2A, at a site which we have predicted to bind directly to RAD51 in the wildtype complex. We have performed an additional MD simulation of the BRC2A S08P mutant in complex with RAD51 and found that, as expected, the S08P mutation significantly reduces the binding free energy of BRC2A by over 10 kcal/mol (Figure S5). Hence, by using atomistic simulations, we are able to directly link pathogenic mutations in the BRC repeats with changes in binding affinity, which may in turn affect the integrity of HDR. Interestingly, the alanine scan (Figure 8(b)) reveals that the source of this reduction in binding free energy is not only the loss of direct interactions between S08 and RAD51, but also the removal of the T12-D187 hydrogen bond. The geometry of the proline mutation does not allow intra-hairpin backbone hydrogen bonds and the idea that destabilisation of the hairpin and loss of co-operativity between residues spanning the hairpin may reduce binding affinity will be investigated further in the next section.

### Hairpin stability is vital in maintaining the interactions between RAD51 and the BRC repeats

We have already proposed that T08 in the BRC4 repeat plays an important role in stabilising the interactions between the 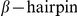 and RAD51, namely the S10-D187 and K12-D187 hydrogen bonds, by forming side chain hydrogen bonds with the side chain of S10 and the backbone of K12 (Figure S2(a)) and hydrophobic contacts with the methylenes of the K12 side chain and the D187 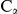 atom (Figure S2(b)). We now investigate this stabilisation further by examining the binding behaviour of BRC6A, which contains the strongly hydrophobic isoleucine residue at position 08.

**Figure 9:**
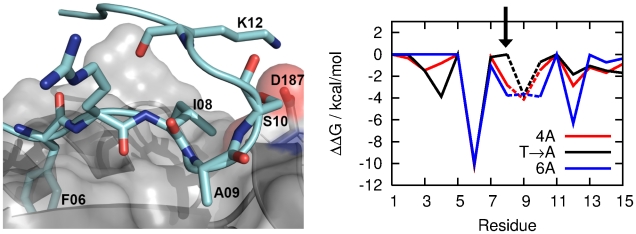
Snapshot of the RAD51-BRC6A interaction and corresponding computational alanine scan. Dashed lines indicate an alanine to glycine mutation. Also shown is the alanine scan of the T08A mutation in RAD51-BRC4A. A less bulky hydrophobic group at position 08 appears to cause reduced contributions to binding at sites 10 and 12.

Figure 9 shows a snapshot of the RAD51-BRC6A interaction and the results of the computational alanine scan. Firstly, the direct hydrophobic interaction between I08 and D187 of RAD51 is increased relative to T08. Interestingly, the T08I substitution has the additional effect of increasing the occupancy of the S10-D187 and K12-D187 hydrogen bonds (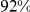 and 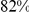 in BRC6A vs. 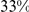 and 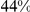 in BRC4A), which can be rationalised by the observation of strong hydrophobic contact between D187, I08 and K12 (Figure S2(b)). Very similar behaviour is observed in MD simulations of the cancer-associated T08I mutation in RAD51-BRC7A (Figure S5). Indeed, the overall binding free energy is actually increased relative to wildtype BRC7A.

Although, intra-hairpin hydrogen bonding interactions (T08-S10 and T08-K12) are lost upon T08I substitution, there is no evidence of this causing a decrease in stabilisation in the RAD51-BRC6A or mutant RAD51-BRC7A interactions. The unexpected relative stability of RAD51-BRC6A in MM-PBSA compared with our FP assays may be due to limitations of the computational model, such as the neglect of the BRC6B C-terminus and the N-terminal domains of RAD51. However, a more likely scenario is that the BRC6A (and mutated BRC7A) hairpin will unfold on time scales longer than we can access in our simulations. Indeed, the intra-hairpin hydrogen bond formed between the backbones of residues 08 and 12 undergoes larger fluctuations in simulations of BRC repeats containing I08 than in any repeats containing the highly-conserved residue T08 (Figure S6), which may result in a shorter lifetime of the 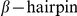 fold and loss of binding free energy over longer time scales [40].

We have shown above that, on the time scale of these simulations, the T08I substitution enhances the contributions of the hairpin residues, S10 and K12, to binding. To investigate whether this binding contribution may be reduced in some circumstances, we have also investigated the T08A mutation in RAD51-BRC4A (a previously identified structural mutation derived from an equivalent cancer-associated mutation in BRC1). In agreement with our speculation that T08 stabilises the S10-D187 and K12-D187 interactions, the alanine scan (Figure 9) reveals reduced contributions to binding from both S10 and K12 upon T08A mutation (Figure S2(b)), as well as loss of direct interactions from residue T08. Despite this clear loss of binding around the hotspot region, MM-PBSA actually predicts the T08A mutant to have a more favourable binding free energy than wildtype BRC4A (Figure S5).

In order to resolve this discrepancy between MM-PBSA and our analysis of the BRC4A binding hotspot, we have sought to also compute the free energy of the T08A mutation in BRC4A by thermodynamic integration (TI) [41], [42]. The TI technique is one of the most rigorous approaches for calculating free energy changes as it actually connects the start and end states of the mutation along a non-physical but thermodynamically well-defined path of intermediate species (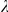 values) in order to calculate the free energy change associated with the transformation. Provided the sampling is converged with respect to the number of 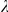 values, it naturally includes all of the entropic contributions from the accessible conformational space, going beyond the harmonic frequencies approximation of the MM-PBSA approach and limited only by the quality of the force field. It is therefore a more rigorous method than the single trajectory MM-PBSA approach, but is also considerably more computationally expensive as one TI calculation will typically need about 100 times more computation than an MM-PBSA calculation. The change in free energy obtained by TI for the T08A mutation in RAD51-BRC4A is 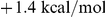. This change in binding free energy is sufficient to explain the weak competitive inhibition of the BRC4 T08A mutant relative to wildtype BRC4 in our FP assay (Figure 1(b)) and confirms that T08 plays an important role in RAD51-BRC4A binding, forming direct contact with RAD51 and maintaining hairpin stability.

## Discussion

We have carried out an investigation of the interactions that determine the stability of a protein-protein system that is essential for normal cellular function (DNA repair) and found to be mis-regulated in cancer. Crucially, this biologically significant protein-protein interaction occurs between a single protein (RAD51) and, mutually exclusively, one of several protein motifs in another (BRCA2) that have measurable variation in affinity despite only subtle changes in sequence. Our approach is based on a combination of computational and experimental techniques and seeks to establish the relative binding affinity of each of the eight human BRC repeats to RAD51.

It has recently been proposed that the BRC repeats interact with RAD51 through two energetic “hotspot” regions, which are in distinct modules of the repeat (termed here, BRCnA and BRCnB) [19]. The reported differences in affinity of a complete BRC repeat for RAD51 are likely to be explained both by the total contributions of both modules to interactions with RAD51, as well as their site accessibility to RAD51 in different functional settings (e.g. monomeric, oligomeric or filamentous forms on DNA). Although each module comprises a discrete tetrameric binding motif with potentially divergent functional effects on RAD51, in this study, we have decided to focus our computational simulations on BRCnA peptides as we have more structural and biochemical insights into the conformations explored by both the BRC repeat and RAD51 components within the confines of this region of the protein-protein interface.

Due to the inherent experimental difficulties with these systems, such as the spontaneous aggregation of purified RAD51 [43], it has not been possible to measure, so far, free energies of binding through approaches such as isothermal titration calorimetry for all eight BRC repeats with a fully monomeric RAD51 core catalytic domain (in accord with the crystallised BRC4-RAD51 complex). We have been able to fill this knowledge gap by using a wide range of computational techniques, not only to measure relative free energies of binding of the different repeats, but also to link these affinities with the variations in sequence observed across the BRC repeats. For systems such as these, for which relatively little experimental structural information is available, we emphasise the need for multiple computational approaches, balancing accuracy with computational expense. Studies employing homology modelling and the computation of *in silico* interaction energies are able to scan a large number of residues at the BRCn-RAD51 interface with high efficiency and have successfully predicted a number of mutations that enhance RAD51-BRC4 binding (e.g. the L1545F mutation in the “LFDE” hotspot) [37]. However, this approach assumes that each of the BRC repeats interacts in the same manner as BRC4 with RAD51 and neglects both relative free energies of solvation of the ligands and longer time scale dynamics of their interaction with RAD51 (such as the S10-D187 hydrogen bonds that fluctuate on nanosecond time scales).

In this paper, we have approached the system with a more rigorous (and also more computationally expensive) set of methods that are applicable across any protein-protein interaction. In using classical MD to sample the conformational space of the complexes derived from the RAD51-BRC4 crystal structure, we have assumed that the BRC repeats interact with binding modes broadly similar to those of BRC4, but also found small but significant differences such as the model we have proposed for the RAD51-BRC2A interface. By post-processing the resultant MD trajectories using MM-PBSA analysis, we have obtained a relative order of binding that is in reasonable qualitative agreement with our FP assays. Furthermore, the QM-PBSA method we have developed allows us to compute binding free energies from large-scale quantum mechanical first principles calculations, which is an important step towards resolving the affinities of similar repeats which typically vary by a few kcal/mol. In order to link these free energy calculations with variations in sequence, a useful tool is alanine scanning mutagenesis, which estimates the contributions of each residue of the BRC repeats to the total binding energy, can be compared directly with experiment and can be used to reveal binding hotspots and potential sites for small molecule targeting. The alanine scanning derived contributions confirm the hotspot model and show that the majority of the binding energy is concentrated in the FxxA hotspot of BRC4A and its analogues for the other repeats. Also, based on computational alanine scanning and significant to the processes behind the regulation of RAD51 by BRCA2, we have rationalised experimental observations that the A09S mutation in BRC4 reduces the free energy of binding to RAD51 [37].

The sequence variation in the BRCnA region has a significant effect on the stability of the structural environment in which the FxxA hotspot is embedded. The hairpin domain of the BRCnA repeats is critical for maintaining potent interaction with RAD51 and we have found that both T08 and I08 are capable of stabilising this fold, on the time scale of these simulations, via intra-hairpin interactions. Stability of the hairpin can be compromised by mutations that are associated with cancer predisposition and may hence compromise the integrity of HDR. The computational tools we have employed allow us the ability to study the effect of essentially any mutation to the repeats and we have hence used them to interpret the mechanism of crucial cancer-associated mutations. For example, alanine scanning mutagenesis reveals reduced inter-protein interactions between RAD51 and the hairpin of the T08A mutant form of BRC4A. In this case, MM-PBSA fails to recover the relative binding affinities of wildtype BRC4 and its mutant form observed in our FP assays and so we have turned to the more accurate and computationally expensive TI technique to confirm our observations from experiment and computational alanine scanning. Simulations such as these are vital since mutations do not always fall in the BRC repeat for which there is a high-resolution structure or in a region of obvious functional relevance in the BRC repeat (we note that the known cancer-associated mutations are not in the F/A residues of FxxA but they do have an effect on it). This may be true of L/F/D/E as well (no known mutations in hotspot residues but the structural context may be affected).

There is significant therapeutic relevance associated with our insights into the behaviour of the 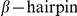 in the context of the natural variation found in different repeats and our understanding of how the interaction with RAD51 can be enhanced or reduced. We envisage that our approach can be used for the rational computer-aided design of peptidomimetic drugs that specifically compete for, and block, the BRC-RAD51 interaction. This could be achieved through mimicry of the BRC-RAD51 interface or potentially through the use of “stabilised hairpins”, in a manner akin to recent developments in the stabilisation of 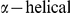 chemical scaffolds [44]. By defining how a promiscuous interface is able to interact with different primary sequences with varying binding capacities, we have the potential to understand the dynamic nature of protein-protein interactions and identify the determinants of molecular discrimination that could be studied with regard to biological consequences of binding mode and mechanism of protein-protein interactions and re-evaluating peptidomimetic insights for the rational design of small molecule targeting of protein-protein interactions.

## Methods

### Fluorescence polarisation assay

FP measurements were carried out in a 384-well, low-volume, black, flat bottom polystyrene NBS microplate (Corning 3820) using a PHERAstar Plus plate reader (BMGLabtech). The polarisation values are reported in millipolarisation units (mP) and were measured at an excitation wavelength of 485 nm and an emission wavelength of 520 nm. Following assay optimisation, full length wild type RAD51 protein was used at a final concentration of 135 nM and Alexa488-BRC4 peptide at 10 nM. By varying the concentration of Alexa488-BRC4 it was shown that FP was independent of total fluorescence (data not shown). The Z′ factor for the assay was calculated to be 0.771 (data not shown). To assess the relative ability of the BRC repeats to displace the Alexa488-BRC4 in this assay, unlabelled BRC repeat peptide was added to each well at a final concentration of 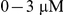 (serial dilution) and measurements made in quadruplicates. To validate the method, the experiment was repeated for selected BRC repeats using an ELISA assay (Figure S7), as described previously by Rajendra and Venkitaraman [19].

### Peptides

All peptides, listed in Figure 3 but with full sequences as described in Figure S8, were synthesised by the Cancer Research UK Peptide Synthesis Facility with a C-terminal amide except Alexa488-BRC4, synthesised by Cambridge Research Biochemicals Ltd with an additional N-terminal Alexa488 moiety attached by an aminohexanoic acid spacer. Peptides were purified to 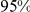 by HPLC, sequence-verified by time-of-flight mass spectrometry and diluted in water.

### Computational

MD simulations were performed with the amber10 package [45], using the X-ray crystal structure of the RAD51-BRC4 complex [18] (PDB: 1N0W) as the starting structure. Water molecules were treated using the TIP3P force field and all protein interactions were described by the amber ff99SB biomolecular force field [46]. Coulomb interactions were treated using the Particle Mesh Ewald sum, with a real space cut-off of 10 Å. The cut-off length for Lennard-Jones interactions was also set to 10 Å. A short energy minimisation was performed in vacuum to remove steric contacts, water and sodium counter-ions were added and the system was heated to 300 K with weak harmonic restraints on the complex at constant pressure (NPT ensemble). Finally, all restraints were removed and the system was equilibrated for 2 ns at 300 K, at the end of which the root mean square deviation of the protein backbone atoms was converged and was less than 2 Å relative to the original crystal structure. In addition, three 12 ns production runs (with different initial velocities) were performed to provide structures for a computational alanine scan of the full RAD51-BRC4 complex (Figure 2(b)).

In order to study the relative binding affinities of the eight BRC repeats to RAD51, we have removed all water molecules from the equilibrated structure of RAD51-BRC4 and truncated the BRC4 peptide to include only the N-terminal 15 residues (P1519-K1533) that bind to the RAD51 oligomerisation interface (RAD51-BRC4A). RAD51 was terminated by 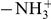 and 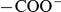 groups, which are more than 25 Å from the hotspot region and are, therefore, not expected to affect binding energetics. The BRC4 half peptides were all terminated by 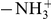 and 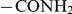 in accordance with our experimental procedures. The choice of terminal groups for the BRC repeats may affect the strength of binding determined by MM-PBSA, as discussed in the results section, but does not affect the contribution of each residue in the hotspot region to binding, on which our discussion concerning sequence variation is based. Starting structures of the remaining seven BRC repeats in complex with RAD51 were obtained by truncating at the 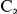 atom only residues that differ between the two structures and using the leap module of amber10 to rebuild the mutated side chains. The resulting eight complexes (plus the five mutated ligands detailed in Figure 3 and discussed in Supporting Text S1) were re-solvated and, as above, were heated to 300 K and simulations were performed for times ranging from 26 ns to 53 ns (simulations were stopped when the running average of the MM-PBSA binding free energy did not vary by more than 1 kcal/mol in the final 12 ns). Snapshots were saved every 6 ps for MM-PBSA single trajectory analysis over the final 24 ns. In order to test the reproducibility of alanine scanning mutagenesis of the hotspot regions, we have performed an additional 24 ns simulation of the RAD51-BRC6A complex (Figure S9).

The above procedure predicted BRC2A to be a weak binder, in contrast to our FP measurements. It may be expected that the RAD51-BRC4A complex is a poor starting configuration since homology with the RAD51-BRC2A complex is relatively low. To further explore the configuration space of BRC2A, an additional simulation was performed starting with the RAD51-BRC2 complex (entire BRC2) and following 2 ns of simulation, the final structure was truncated and used as input for the RAD51-BRC2A simulation, which led to the reported dynamics and a favourable binding free energy. To confirm that this approach was not artificially lowering the binding free energy, a similar procedure was applied to the RAD51-BRC3A interaction. No gain in binding free energy was observed.

Precluding computational analyses on the oligomerisation interface between RAD51 monomers (competed by the BRCnA region of the BRC repeats), no high-resolution crystal structure exists for a human RAD51 oligomeric species either in solution or on DNA. In order to address this problem, we performed analyses on a modelled structure based on the interface between Rad51 protomers from the budding yeast *Saccharomyces cerevisiae* Rad51 orthologue [33] (PDB: 1SZP). Both the “receptor” and “ligand” components of a dimeric unit of the ScRad51 dimer were truncated to match as closely as possible the complexes between the humanised RAD51 receptors and the BRC repeats described above. Namely, the Rad51 receptor N-terminus was removed, keeping only residues E156-P395. In order to compare directly with the BRCnA peptides, a 15 amino acid peptide (L139-R153), which is responsible for binding at the Rad51-Rad51 interface in yeast and contains the FVTA motif (conforming with the FxxA motif conserved across RAD51 orthologues), was retained as the ligand. The truncated complex was minimised in vacuum and equilibrated in water at 300 K, as in Figure 2(a). Finally, the equilibrated complex was removed from water and all residues of both the receptor and ligand were mutated to the human form, leaving a 15 amino acid ligand containing the FTTA motif interacting with the fully “humanised” RAD51 receptor. The root mean square deviation of the backbone atoms of the resulting complex remained below 2.5 Å relative to the equilibrated yeast structure throughout the subsequent production run, indicating that the yeast dimer is a reasonable input model for human RAD51.

Although yeast have no identifiable BRCA2 orthologue and yeast filament structures have been shown to differ slightly from those of human RAD51 by low resolution electronic microscopic reconstruction [47], [48], our method of humanisation and energy minimisation of the yeast structure make this model suitable for our analyses. Furthermore, we have based our model on the closest orthologue of human RAD51 that is currently available with a high-resolution structure, and the I345T mutation used to aid crystallisation of the yeast Rad51 on DNA [33] is not expected to affect the oligomerisation interface studied here.

Free energy calculations of the resulting trajectories were performed using both MM-PBSA [24] and QM-PBSA [23] techniques, retaining 163 residues of the RAD51 receptor (a 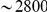 atom complex). Classical free energy calculations were carried out using the MM-PBSA post-processing module in amber10. In the single trajectory MM-PBSA approach, the relative free energy of binding between a receptor and its ligands is given by:
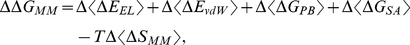
(1)where the gas phase binding energy is split into electrostatic (EL) and van der Waals (vdW) terms, and averaged over the ensemble of snapshots extracted from the MD simulation. Infinite non-bonded cut-offs were used for these molecular mechanics contributions. Similarly, the binding free energy of solvation from the Poisson-Boltzmann continuum solvation model includes electrostatic (PB) and non-polar surface area (SA) terms. For calculating the free energy of solvation, dielectric constants of 1.0 and 80.0 were used for the solute and solvent respectively and the Poisson-Boltzmann equation was solved on a grid of spacing 0.5 Å. A spherical solvent probe of radius 1.4 Å and atomic radii provided by the amber force field were used for the implicit solvent molecules and solute atoms, respectively. The non-polar contribution to the free energy was calculated via 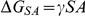, where SA is the solvent-accessible surface area and 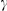 is 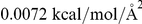. Finally, 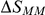 is the binding entropy of the molecules, arising from changes in the translational, rotational and vibrational degrees of freedom of the solute species, and was estimated by normal mode analysis, using the NAB module of amber10. The trajectory was sampled every 0.75 ns and each snapshot was minimised in the generalised Born implicit solvent model, using initially conjugate gradients and then Newton-Raphson minimisation, until the root mean square of the elements of the gradient vector was less than 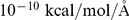. The harmonic frequencies of the vibrational modes were then calculated at 300 K for these minimised structures using normal mode analysis.

Trajectories were sampled every 120 ps for computational alanine scanning using the MM-PBSA post-processing module in amber10. Alanine mutant structures were generated by truncating each residue of the ligand in turn at the 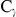 atom and by replacing the 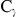 atom with a hydrogen atom at the correct distance along the 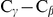 bond. Glycine scans on alanine residues found in the interaction hotspots were performed in the same way by truncating at the 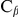 atom, as is standard in alanine scanning experiments [49]. Although glycine scanning cannot be quantitatively compared to alanine scanning, it allows us to compare the contribution to binding of alanine residues on different BRC repeats and qualitatively identifies residues involved in hotspot mediated interactions (Figure S10).

The T08A mutation in RAD51-BRC4A was investigated using TI in amber10. Gaussian quadrature with nine nodes (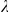) and soft-core potentials [50] were used to smoothly mutate all side chain atoms from threonine to alanine in three stages. At each value of 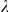, the system was minimised for 1000 steps and heated to 300 K over a period of 0.15 ns with restraints on the heavy atoms of the proteins. To avoid large temperature fluctuations in the solute, a Langevin thermostat with a collision frequency of 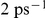 was employed with a time step of 1 fs. All restraints were removed and the systems were equilibrated for periods ranging from 2 to 6 ns. Productions runs lasted from 2.5 to 8 ns, with the vdW transformations requiring longer simulations to reach convergence (Figure S11).

In the QM-PBSA approach [23], the relative free energies of binding are replaced by:
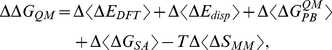
(2)where instead of using a classical force field to obtain the gas phase binding energy of each snapshot, we use a full DFT quantum mechanical calculation. Quantum mechanical calculations of total energies were performed with the onetep program [51], using the PBE gradient corrected exchange-correlation functional [52]. Interactions between electrons and nuclei were described by norm-conserving pseudopotentials. The onetep program achieves computational cost that scales linearly with the number of atoms by exploiting the “near-sightedness” of the single-particle density matrix in non-metallic systems [53]. The density matrix is expressed in terms of a set of non-orthogonal generalised Wannier functions (NGWFs) [54] that are localised in real space with radii of 4.0 Å. The NGWFs were expanded in a basis of periodic cardinal sine (psinc) functions [55] with a kinetic energy cut-off of 830 eV. The spherical cut-off approach for Coulomb potentials [56] was used to eliminate all interactions of the molecules with their periodic images. Van der Waals interactions were included by augmenting the DFT energy expression by damped London potentials with parameters optimised specifically for the PBE functional [57] (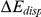). The root mean square error in gas phase binding energies of a benchmark set of complexes calculated using the DFT methodology described above has been shown to be approximately 1 kcal/mol when compared to MP2 and CCSD(T) methods extrapolated to the complete basis set limit [57]. 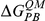 is the weighted polar part of the solvation free energy from the MM calculation:
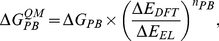
(3)where 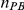 is determined for each complex studied by a best fit power law curve to a plot of 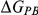 against 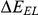 [23]. QM-PBSA is more computationally demanding than MM-PBSA and so the trajectory was sampled every 1.5 ns. Following previous work [23], in order to improve convergence with the number of snapshots sampled, four additional snapshots were chosen so as to minimise the difference between the properties of the sampled set (as calculated by MM) and the high sample limit of the MM distribution. The chosen properties were the mean and standard deviation of the binding free energy and the fractional occupancies of intermittent hydrogen bonds (D187-S10 and D187-K12 for the repeats BRC1A, BRC4A and BRC6A and D187-T10 for RAD51-RAD51). Using this method, total binding free energies were converged to within 0.5 kcal/mol with respect to the number of snapshots sampled.

MM force errors were evaluated as the magnitude of the vector joining the MM and QM forces for each atom, 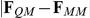, averaged over snapshots sampled every 1.5 ns.

## Supporting Information

Figure S1 **Binding modes of the LFDE and WLRE hotspots in BRC4B and BRC5B.** (top) The LFDE binding hotspot in the geometry of the 1N0W crystal structure. The backbone of F1546 forms a hydrogen bond with R250. (bottom) In simulations of the RAD51-BRC5B complex lacking the RAD51 N-terminus, the BRC5B C-terminus interferes with binding and R250 moves away from the hotspot leading to a low binding free energy.
(PDF)

Figure S2 **Non-bonded contacts between selected residues in the BRC hairpin.** Block averages of non-bonded interactions involving residue 08. a) Residue T08 in BRC4A forms intra-hairpin hydrogen bonds with residues S10 and K12, which are important in maintaining hairpin stability. The corresponding interaction in RAD51 (T08–T10) is less important and fluctuates throughout the simulation. b) T08 also forms hydrophobic contacts with D187 of RAD51 and K12, which may help to stabilise the D187-K12 hydrogen bond. This effect is enhanced following the T08I substitution and reduced in T08A. Dashed lines indicate the corresponding distances in 1N0W.
(PDF)

Figure S3 **Computational alanine scans of BRC1A, BRC3A, BRC7A and BRC8A.** Computational alanine scans of the BRC4A-like repeats. All show very similar profiles close to the FxTA binding hotspot (residues 06–09) and binding affinity is instead determined by the overall charge of each repeat.
(PDF)

Figure S4 **Selected hydrogen bond lengths in BRCnA-RAD51 complexes.** Block averages of two backbone inter-protein hydrogen bonds (solid and dashed lines) in simulations of the interaction between RAD51 and the BRC repeats, compared to the 1N0W crystal structure. The backbone hydrogen bonds are longer in the BRC2A interaction than in the BRC4A interaction, which may be a result of the different binding modes observed.
(PDF)

Figure S5 **Relative binding free energies of five mutated BRC repeats.** a) Binding free energy of each of the single residue cancer-associated mutations studied, relative to the corresponding wildtype BRC repeat. As discussed in the main text, mutation in the hotspot region of BRC2A causes a decrease in binding free energy. The T08A mutation in BRC4A causes a net gain in binding free energy despite the loss of binding observed in an alanine scan of the hotspot region. b) The alanine scan of the BRC7A T08I mutation is similar to that of the RAD51-BRC6A interaction. Alanine scans of c) the BRC4A G11R and d) the BRC1A T08R mutations, which are discussed in Supplementary Text S1.
(PDF)

Figure S6 **Distribution of a backbone hydrogen bond in residues containing the T08I mutation.** Logarithmic distribution of the backbone hydrogen bond formed between residues 08 and 12, across the hairpin, in four different BRC repeats. All BRC repeats containing T08 (e.g. BRC4A and BRC7A) have very similar distributions, and do not fluctuate beyond 3.5 Å. In BRC6A and mutated BRC7A, both of which contain the T08I substitution, fluctuations are more pronounced, which may lead to unfolding of the hairpin on very long time scales.
(PDF)

Figure S7 **ELISA assay.** An ELISA assay demonstrates inhibition of the BRC4-RAD51 interaction by the T1526A peptide. RAD51, bound to a BRC4 peptide in the solid phase, was detected using a rabbit polyclonal antibody against RAD51. Disruption of this interaction, using soluble BRC peptides (BRC4, BRC4-T1526A and BRC5), caused a reduction in the colorimetric change induced by the action of an HRP-conjugated anti-rabbit secondary antibody on the substrate 3, 3′, 5, 5′- tetramethylbenzidine.
(PDF)

Figure S8 **Sequence alignment of the BRC repeats used in FP assays.** Sequence alignment of the eight human BRC repeats and a BRC4T08A mutation used in our FP assays, generated with ClustalW. The symbols on the bottom row denote the degree of conservation observed in each column: ‘*’ denotes that the residues in that column are identical in all sequences in the alignment, ‘:’ denotes that conserved substitutions have been observed and ‘.’ denotes that semi-conserved substitutions are observed.
(PDF)

Figure S9 **Reproducibility of alanine scanning for the RAD51-BRC6A interaction.** Two computational alanine scans of the RAD51-BRC6A interface (grey), showing the reproducibility of the residues that contribute most to binding in long simulations.
(PDF)

Figure S10 **Glycine scan of the RAD51-BRC4A interface.** Comparison between computational glycine scan and computational alanine scan of the RAD51-BRC4A interface. The differences between the two methods are small but may be significant around the hotspot region.
(PDF)

Figure S11
**Convergence of thermodynamic integration results.** Convergence of the free energy of the T08A mutation in the RAD51-BRC4A complex using thermodynamic integration in three stages. Stage 1: Removal of partial charges from T08, stage 2: transformation of vdW parameters of T08 to A08 using soft-core potentials, stage 3: introduction of partial charges to A08. 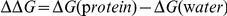. T08 does not form any direct hydrogen bonds with RAD51, yet the majority of the free energy change is due to the removal of charge in stage 1.(PDF)

Text S1 **Additional MM-PBSA simulations of cancer-associated BRC mutants.**
(PDF)

## References

[1] Thorslund T, West SC (2007). Brca2: a universal recombinase regulator.. Oncogene.

[2] Moynahan ME, Jasin M (2010). Mitotic homologous recombination maintains genomic stability and suppresses tumorigenesis.. Nat Rev Mol Cell Biol.

[3] Lo T, Pellegrini L, Venkitaraman AR, Blundell TL (2003). Sequence fingerprints in brca2 and rad51: implications for dna repair and cancer.. DNA Repair (Amst).

[4] Bignell G, Micklem G, Stratton MR, Ashworth A, Wooster R (1997). The brc repeats are conserved in mammalian brca2 proteins.. Hum Mol Genet.

[5] Shivji MK, Mukund SR, Rajendra E, Chen S, Short JM (2009). The brc repeats of human brca2 differentially regulate rad51 binding on single- versus double-stranded dna to stimulate strand exchange.. Proc Natl Acad Sci USA.

[6] Carreira A, Hilario J, Amitani I, Baskin RJ, Shivji MK (2009). The brc repeats of brca2 modulate the dna-binding selectivity of rad51.. Cell.

[7] Esashi F, Galkin VE, Yu X, Egelman EH, West SC (2007). Stabilization of rad51 nucleoprotein filaments by the c-terminal region of brca2.. Nat Struct Mol Biol.

[8] Davies OR, Pellegrini L (2007). Interaction with the brca2 c terminus protects rad51-dna filaments from disassembly by brc repeats.. Nat Struct Mol Biol.

[9] Ayoub N, Rajendra E, Su X, Jeyasekharan AD, Mahen R (2009). The carboxyl terminus of brca2 links the disassembly of rad51 complexes to mitotic entry.. Curr Biol.

[10] Moynahan ME, Pierce AJ, Jasin M (2001). Brca2 is required for homology-directed repair of chromosomal breaks.. Mol Cell.

[11] Patel KJ, Yu VP, Lee H, Corcoran A, Thistlethwaite FC (1998). Involvement of brca2 in dna repair.. Mol Cell.

[12] Shivji MK, Davies OR, Savill JM, Bates DL, Pellegrini L (2006). A region of human brca2 containing multiple brc repeats promotes rad51-mediated strand exchange.. Nucleic Acids Res.

[13] San Filippo J, Chi P, Sehorn MG, Etchin J, Krejci L (2006). Recombination mediator and rad51 targeting activities of a human brca2 polypeptide.. J Biol Chem.

[14] Saeki H, Siaud N, Christ N, Wiegant WW, van Buul PPW (2006). Suppression of the dna repair defects of brca2-deficient cells with heterologous protein fusions.. Proc Natl Acad Sci USA.

[15] Thorslund T, McIlwraith MJ, Compton SA, Lekomtsev S, Petronczki M (2010). The breast cancer tumor suppressor brca2 promotes the specific targeting of rad51 to single-stranded dna.. Nat Struct Mol Biol.

[16] Jensen RB, Carreira A, Kowalczykowski SC (2010). Purified human brca2 stimulates rad51-mediated recombination.. Nature.

[17] Liu J, Doty T, Gibson B, Heyer WD (2010). Human brca2 protein promotes rad51 filament formation on rpa-covered single-stranded dna.. Nat Struct Mol Biol.

[18] Pellegrini L, Yu DS, Lo T, Anand S, Lee M (2002). Insights into dna recombination from the structure of a rad51-brca2 complex.. Nature.

[19] Rajendra E, Venkitaraman AR (2010). Two modules in the brc repeats of brca2 mediate structural and functional interactions with the rad51 recombinase.. Nucl Acids Res.

[20] Chen CF, Chen PL, Zhong Q, Sharp ZD, Lee WH (1999). Expression of brc repeats in breast cancer cells disrupts the brca2-rad51 complex and leads to radiation hypersensitivity and loss of g2/m checkpoint control.. J Biol Chem.

[21] Wong AKC, Pero R, Ormonde PA, Tavtigian SV, Bartel PL (1997). Rad51 interacts with the evolutionarily conserved brc motifs in the human breast cancer susceptibility gene brca2.. J Biol Chem.

[22] Thorslund T, Esashi F, West SC (2007). Interactions between human brca2 protein and the meiosisspecific recombinase dmc1.. EMBO J.

[23] Cole DJ, Skylaris CK, Rajendra E, Venkitaraman AR, Payne MC (2010). Protein-protein interactions from linear-scaling first-principles quantum-mechanical calculations.. EPL.

[24] Massova I, Kollman PA (1999). Computational alanine scanning to probe protein-protein interactions: A novel approach to evaluate binding free energies.. J Am Chem Soc.

[25] Chen PL, Chen CF, Chen Y, Xiao J, Sharp ZD (1998). The brc repeats in brca2 are critical for rad51 binding and resistance to methyl methanesulfonate treatment.. Proc Natl Acad Sci USA.

[26] Bradshaw RT, Patel BH, Tate EW, Leatherbarrow RJ, Gould IR (2011). Comparing experimental and computational alanine scanning techniques for probing a prototypical protein-protein interaction.. Protein Eng Des Sel.

[27] Srinivasan J, Cheatham TE, Cieplak P, Kollman PA, Case DA (1998). Continuum solvent studies of the stability of dna, rna, and phosphoramidate–dna helices.. J Am Chem Soc.

[28] Gohlke H, Case DA (2004). Converging free energy estimates: Mm-pb(gb)sa studies on the proteinprotein complex ras-raf.. J Comput Chem.

[29] Clackson T, Wells JA (1995). A hot-spot of binding-energy in a hormone-receptor interface.. Science.

[30] Clackson T, Ultsch MH, Wells JA, de Vos AM (1998). Structural and functional analysis of the 1 : 1 growth hormone : receptor complex reveals the molecular basis for receptor affinity.. J Mol Biol.

[31] Keskin O, Ma BY, Nussinov R (2005). Hot regions in protein-protein interactions: The organization and contribution of structurally conserved hot spot residues.. J Mol Biol.

[32] Whitty A, Kumaravel G (2006). Between a rock and a hard place?. Nature Chem Biol.

[33] Conway AB, Lynch TW, Zhang Y, Fortin GS, Fung CW (2004). Crystal structure of a rad51 filament.. Nat Struct Mol Biol.

[34] Maurer P, Laio A, Hugosson HW, Colombo MC, Rothlisberger U (2007). Automated parametrization of biomolecular force fields from quantum mechanics/molecular mechanics (qm/mm) simulations through force matching.. J Chem Theory Comput.

[35] Robinson M, Haynes PD (2010). Dynamical effects in ab initio nmr calculations: classical force fields fitted to quantum forces.. J Chem Phys.

[36] Buis N, Skylaris CK, Grant GH, Rajendra E, Payne MC (2008). Classical molecular dynamics simulations of the complex between the rad51 protein and the brc hairpin loops of the brca2 protein.. Mol Sim.

[37] Nomme J, Renodon-Cornière A, Asanomi Y, Sakaguchi Y, Stasiak AZ (2010). Design of potent inhibitors of human rad51 recombinase based on brc motifs of brca2 protein: modeling and experimental validation of a chimera peptide.. J Med Chem.

[38] Wooster R, Neuhausen SL, Mangion J, Quirk Y, Ford D (1994). Localization of a breast cancer susceptibility gene, brca2, to chromosome 13q12-13.. Science.

[39] Wooster R, Bignell G, Lancaster J, Swift S, Seal S (1995). Identification of the breast cancer susceptibility gene brca2.. Nature.

[40] Kieseritzky G, Morra G, Knapp EW (2006). Stability and fluctuations of amide hydrogen bonds in a bacterial cytochrome c: a molecular dynamics study.. J Biol Inorg Chem.

[41] Deng Y, Roux B (2009). Computations of standard binding free energies with molecular dynamics simulations.. J Phys Chem B.

[42] Michel J, Essex JW (2010). Prediction of protein-ligand binding affinity by free energy simulations: assumptions, pitfalls and expectations.. J Comput Aided Mol Des.

[43] Benson FE, Stasiak A, West SC (1994). Purification and characterization of the human rad51 protein, an analogue of e. coli reca.. Embo J.

[44] Walensky LD, Kung AL, Escher I, Malia TJ, Barbuto S (2004). Activation of apoptosis in vivo by a hydrocarbon-stapled bh3 helix.. Science.

[45] Case DA, Darden TA, Cheatham TE, Simmerling CL, Wang J (2008).

[46] Hornak V, Abel R, Okur A, Strockbine B, Roitberg A (2006). Comparison of multiple amber force fields and development of improved protein backbone parameters.. Proteins.

[47] Yu X, Jacobs SA, West SC, Ogawa T, Egelman EH (2001). Domain structure and dynamics in the helical filaments formed by reca and rad51 on dna.. Proc Natl Acad Sci USA.

[48] Sheridan SD, Yu X, Roth R, Heuser JE, Sehorn MG (2008). A comparative analysis of dmc1 and rad51 nucleoprotein filaments.. Nucl Acids Res.

[49] Hanna M, Brault M, Kwan T, Kast C, Gros P (1996). Mutagenesis of transmembrane domain 11 of p-glycoprotein by alanine scanning.. Biochemistry.

[50] Steinbrecher T, Mobley DL, Case DA (2007). Nonlinear scaling schemes for lennard-jones interactions in free energy calculations.. J Chem Phys.

[51] Skylaris CK, Haynes PD, Mostofi AA, Payne MC (2005). Introducing onetep: Linear-scaling density functional simulations on parallel computers.. J Chem Phys.

[52] Perdew JP, Burke K, Ernzerhof M (1996). Generalized gradient approximation made simple.. Phys Rev Lett.

[53] Prodan E, Kohn W (2005). Nearsightedness of electronic matter.. Science.

[54] Skylaris CK, Mostofi AA, Haynes PD, Dieguez O, Payne MC (2002). The non-orthogonal generalized wannier function pseudopotential plane-wave method.. Phys Rev B.

[55] Mostofi AA, Haynes PD, Skylaris CK, Payne MC (2003). Preconditioned iterative minimisation for linear-scaling electronic structure calculations.. J Chem Phys.

[56] Jarvis MR, White ID, Godby RW, Payne MC (1997). Supercell technique for total-energy calculations of finite charged and polar systems.. Phys Rev B.

[57] Hill Q, Skylaris CK (2009). Including dispersion interactions in the onetep program for linear-scaling density functional theory calculations.. Proc R Soc A.

