# Supplementary material for: Interrogation of the Protein-Protein Interactions between Human BRCA2 BRC Repeats and RAD51 Reveals Atomistic Determinants of Affinity
Source: PLoS Comput Biol. 2011 Jul 14;7(7):e1002096. doi: 10.1371/journal.pcbi.1002096 (PMC3136434; doi:10.1371/journal.pcbi.1002096)
Supplement: Figure S1 — Binding modes of the LFDE and WLRE hotspots in BRC4B and BRC5B. (top) The LFDE binding hotspot in the geometry of the 1N0W crystal structure. The backbone of F1546 forms a hydrogen bond with R250. (bottom) In simulations of the RAD51-BRC5B complex lacking the RAD51 N-terminus, the BRC5B C-terminus interferes with binding and R250 moves away from the hotspot leading to a low binding free energy. (PDF) [file pcbi.1002096.s001.pdf]

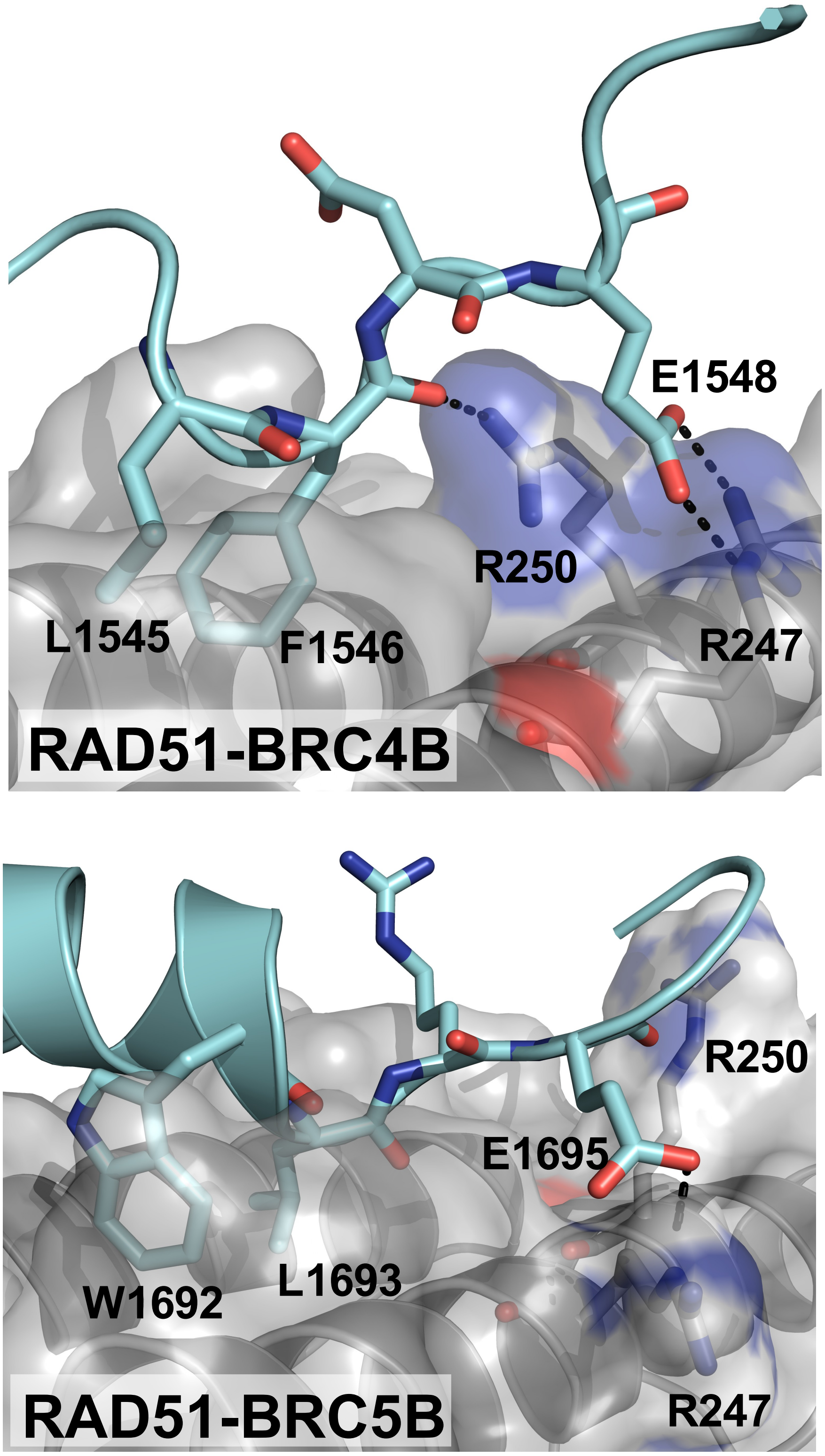

Figure S1. (top) The LFDE binding hotspot in the geometry of the 1N0W crystal structure. The backbone of F1546 forms a hydrogen bond with R250. (bottom) In simulations of the RAD51-BRC5B complex lacking the RAD51 N-terminus, the BRC5B C-terminus interferes with binding and R250 moves away from the hotspot leading to a low binding free energy.
