# Supplementary material for: Interrogation of the Protein-Protein Interactions between Human BRCA2 BRC Repeats and RAD51 Reveals Atomistic Determinants of Affinity
Source: PLoS Comput Biol. 2011 Jul 14;7(7):e1002096. doi: 10.1371/journal.pcbi.1002096 (PMC3136434; doi:10.1371/journal.pcbi.1002096)
Supplement: Figure S2 — Non-bonded contacts between selected residues in the BRC hairpin. Block averages of non-bonded interactions involving residue 08. a) Residue T08 in BRC4A forms intra-hairpin hydrogen bonds with residues S10 and K12, which are important in maintaining hairpin stability. The corresponding interaction in RAD51 (T08–T10) is less important and fluctuates throughout the simulation. b) T08 also forms hydrophobic contacts with D187 of RAD51 and K12, which may help to stabilise the D187-K12 hydrogen bond. This effect is enhanced following the T08I substitution and reduced in T08A. Dashed lines indicate the corresponding distances in 1N0W. (PDF) [file pcbi.1002096.s002.pdf]

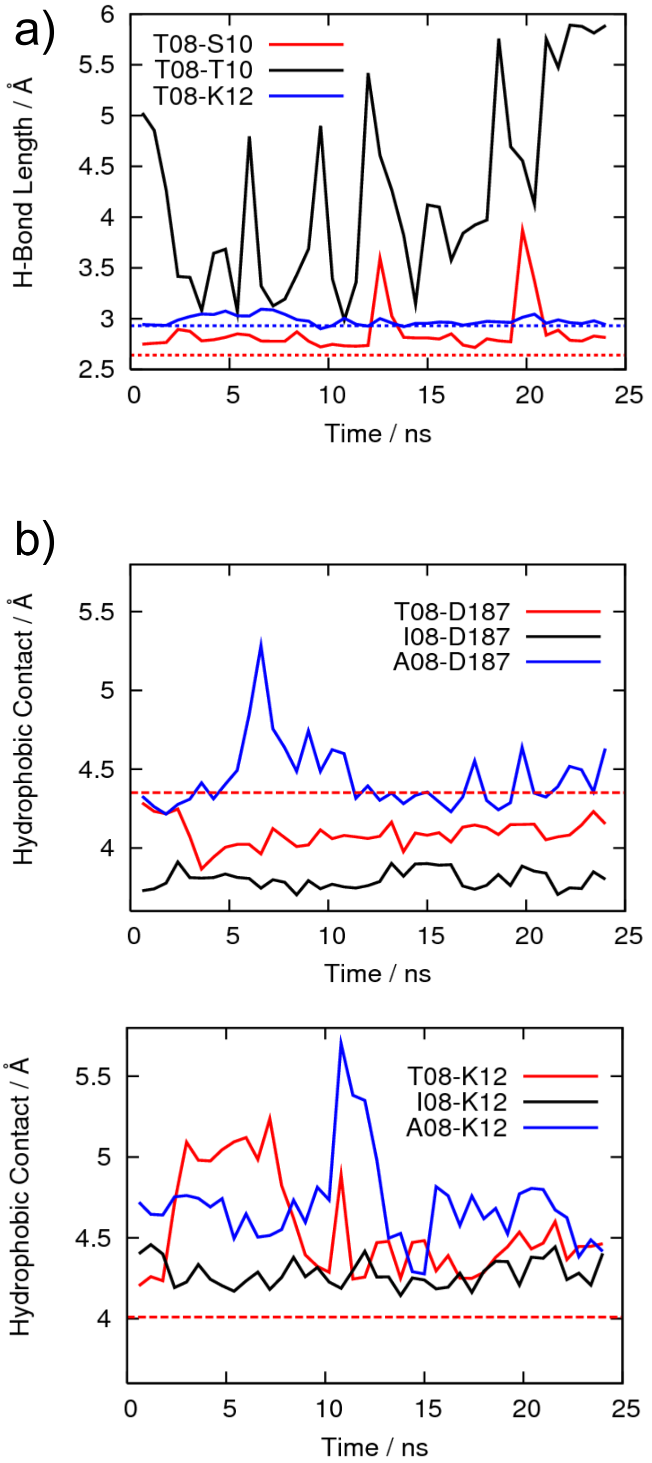

Figure S2. Block averages of non-bonded interactions involving residue 08. a) Residue T08 in BRC4A forms intra-hairpin hydrogen bonds with residues S10 and K12, which are important in maintaining hairpin stability. The corresponding interaction in RAD51 (T08-T10) is less important and fluctuates throughout the simulation. b) T08 also forms hydrophobic contacts with D187 of RAD51 and K12, which may help to stabilise the D187-K12 hydrogen bond. This effect is enhanced following the T08I substitution and reduced in T08A. Dashed lines indicate the corresponding distances in 1N0W.
