# Supplementary material for: Interrogation of the Protein-Protein Interactions between Human BRCA2 BRC Repeats and RAD51 Reveals Atomistic Determinants of Affinity
Source: PLoS Comput Biol. 2011 Jul 14;7(7):e1002096. doi: 10.1371/journal.pcbi.1002096 (PMC3136434; doi:10.1371/journal.pcbi.1002096)
Supplement: Figure S3 — Computational alanine scans of BRC1A, BRC3A, BRC7A and BRC8A. Computational alanine scans of the BRC4A-like repeats. All show very similar profiles close to the FxTA binding hotspot (residues 06–09) and binding affinity is instead determined by the overall charge of each repeat. (PDF) [file pcbi.1002096.s003.pdf]

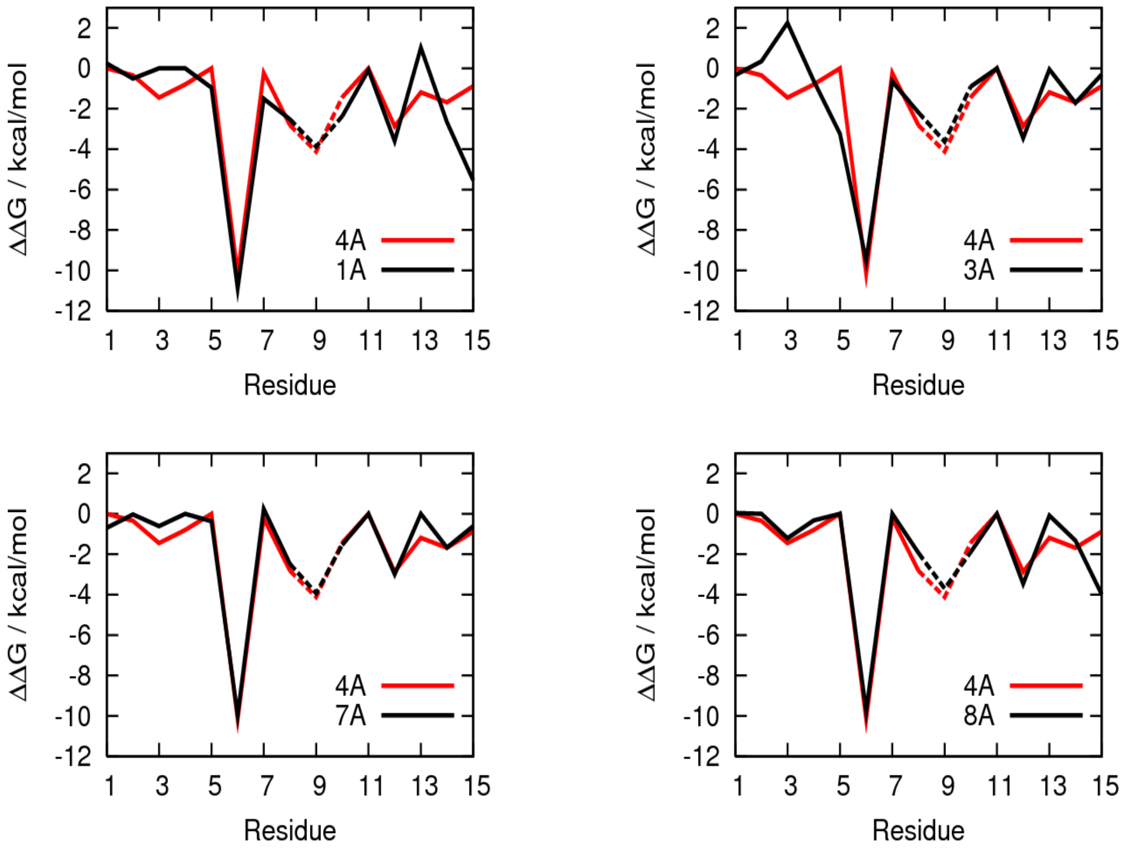

Figure S3. Computational alanine scans of the BRC4A-like repeats. All show very similar profiles close to the FxTA binding hotspot (residues 06-09) and binding affinity is instead determined by the overall charge of each repeat.
