# Supplementary material for: Interrogation of the Protein-Protein Interactions between Human BRCA2 BRC Repeats and RAD51 Reveals Atomistic Determinants of Affinity
Source: PLoS Comput Biol. 2011 Jul 14;7(7):e1002096. doi: 10.1371/journal.pcbi.1002096 (PMC3136434; doi:10.1371/journal.pcbi.1002096)
Supplement: Figure S4 — Selected hydrogen bond lengths in BRCnA-RAD51 complexes. Block averages of two backbone inter-protein hydrogen bonds (solid and dashed lines) in simulations of the interaction between RAD51 and the BRC repeats, compared to the 1N0W crystal structure. The backbone hydrogen bonds are longer in the BRC2A interaction than in the BRC4A interaction, which may be a result of the different binding modes observed. (PDF) [file pcbi.1002096.s004.pdf]

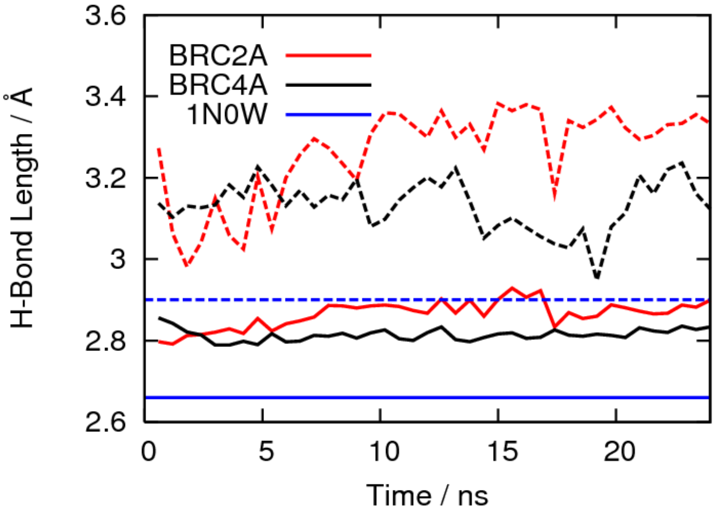

Figure S4. Block averages of two backbone inter-protein hydrogen bonds (solid and dashed lines) in simulations of the interaction between RAD51 and the BRC repeats, compared to the 1N0W crystal structure. The backbone hydrogen bonds are longer in the BRC2A interaction than in the BRC4A interaction, which may be a result of the different binding modes observed.
