# Supplementary material for: Interrogation of the Protein-Protein Interactions between Human BRCA2 BRC Repeats and RAD51 Reveals Atomistic Determinants of Affinity
Source: PLoS Comput Biol. 2011 Jul 14;7(7):e1002096. doi: 10.1371/journal.pcbi.1002096 (PMC3136434; doi:10.1371/journal.pcbi.1002096)
Supplement: Figure S5 — Relative binding free energies of five mutated BRC repeats. a) Binding free energy of each of the single residue cancer-associated mutations studied, relative to the corresponding wildtype BRC repeat. As discussed in the main text, mutation in the hotspot region of BRC2A causes a decrease in binding free energy. The T08A mutation in BRC4A causes a net gain in binding free energy despite the loss of binding observed in an alanine scan of the hotspot region. b) The alanine scan of the BRC7A T08I mutation is similar to that of the RAD51-BRC6A interaction. Alanine scans of c) the BRC4A G11R and d) the BRC1A T08R mutations, which are discussed in Supplementary Text S1. (PDF) [file pcbi.1002096.s005.pdf]

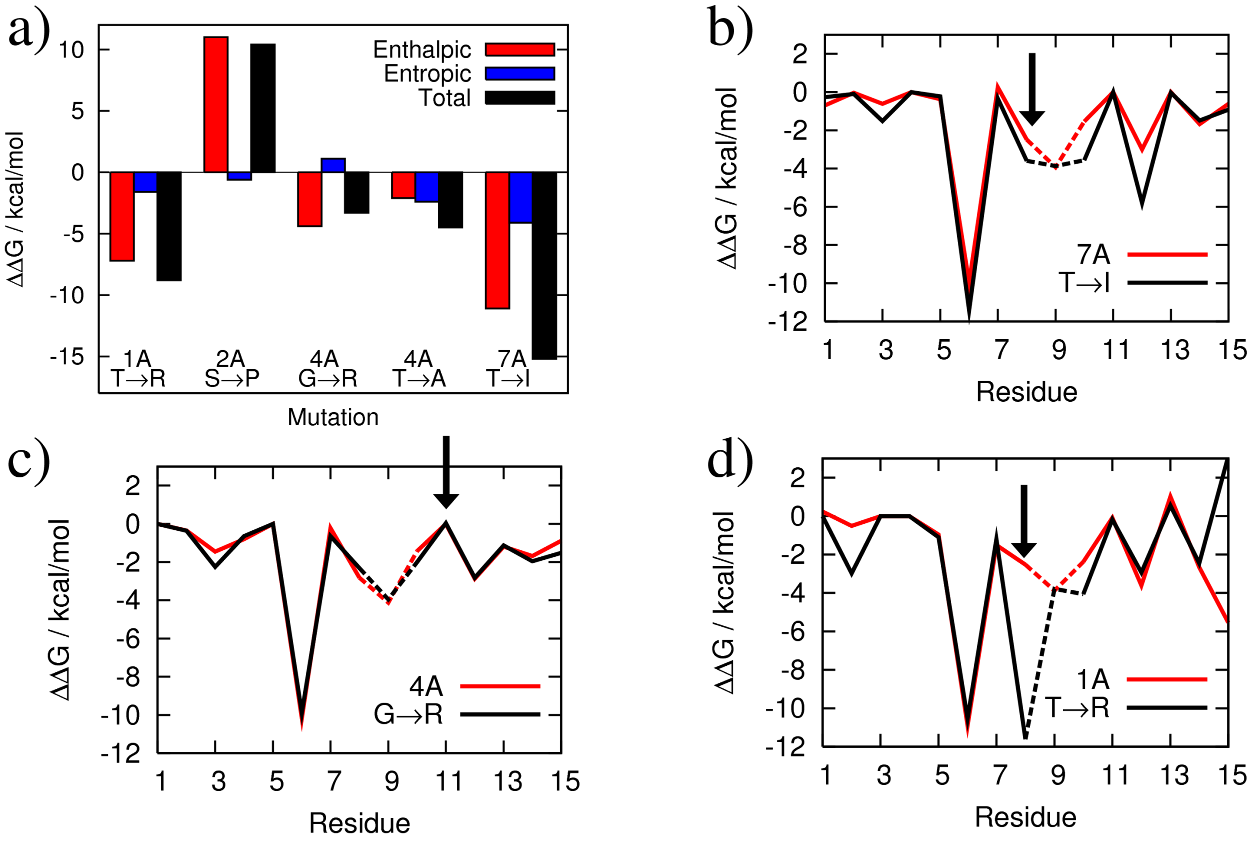

Figure S5. a) Binding free energy of each of the single residue cancer-associated mutations studied, relative to the corresponding wildtype BRC repeat. As discussed in the main text, mutation in the hotspot region of BRC2A causes a decrease in binding free energy. The T08A mutation in BRC4A causes a net gain in binding free energy despite the loss of binding observed in an alanine scan of the hotspot region. b) The alanine scan of the BRC7A T08I mutation is similar to that of the RAD51-BRC6A interaction. Alanine scans of c) the BRC4A G11R and d) the BRC1A T08R mutations, which are discussed in Supplementary Text S1.
