# Supplementary material for: Interrogation of the Protein-Protein Interactions between Human BRCA2 BRC Repeats and RAD51 Reveals Atomistic Determinants of Affinity
Source: PLoS Comput Biol. 2011 Jul 14;7(7):e1002096. doi: 10.1371/journal.pcbi.1002096 (PMC3136434; doi:10.1371/journal.pcbi.1002096)
Supplement: Figure S6 — Distribution of a backbone hydrogen bond in residues containing the T08I mutation. Logarithmic distribution of the backbone hydrogen bond formed between residues 08 and 12, across the hairpin, in four different BRC repeats. All BRC repeats containing T08 (e.g. BRC4A and BRC7A) have very similar distributions, and do not fluctuate beyond 3.5 Å. In BRC6A and mutated BRC7A, both of which contain the T08I substitution, fluctuations are more pronounced, which may lead to unfolding of the hairpin on very long time scales. (PDF) [file pcbi.1002096.s006.pdf]

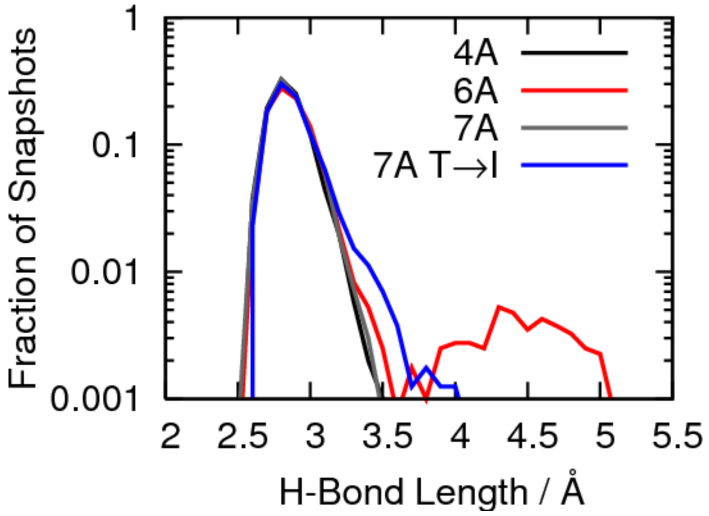

Figure S6. Logarithmic distribution of the backbone hydrogen bond formed between residues 08 and 12, across the hairpin, in four different BRC repeats. All BRC repeats containing T08 (e.g. BRC4A and BRC7A) have very similar distributions, and do not fluctuate beyond 3.5Å. In BRC6A and mutated BRC7A, both of which contain the T08I substitution, fluctuations are more pronounced, which may lead to unfolding of the hairpin on very long time scales.
