# Supplementary material for: Interrogation of the Protein-Protein Interactions between Human BRCA2 BRC Repeats and RAD51 Reveals Atomistic Determinants of Affinity
Source: PLoS Comput Biol. 2011 Jul 14;7(7):e1002096. doi: 10.1371/journal.pcbi.1002096 (PMC3136434; doi:10.1371/journal.pcbi.1002096)
Supplement: Figure S7 — ELISA assay. An ELISA assay demonstrates inhibition of the BRC4-RAD51 interaction by the T1526A peptide. RAD51, bound to a BRC4 peptide in the solid phase, was detected using a rabbit polyclonal antibody against RAD51. Disruption of this interaction, using soluble BRC peptides (BRC4, BRC4-T1526A and BRC5), caused a reduction in the colorimetric change induced by the action of an HRP-conjugated anti-rabbit secondary antibody on the substrate 3, 3′, 5, 5′- tetramethylbenzidine. (PDF) [file pcbi.1002096.s007.pdf]

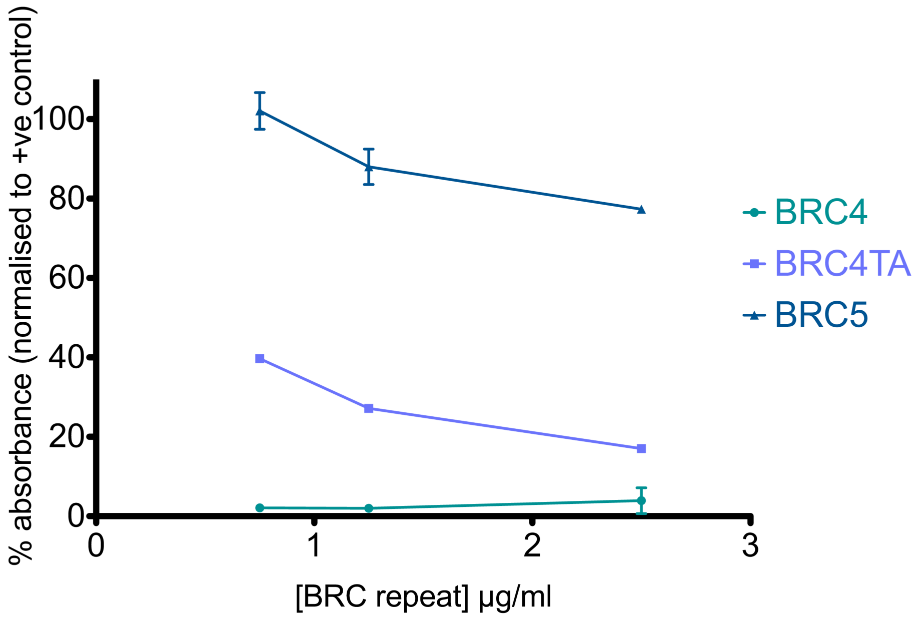

Figure S7. An ELISA assay demonstrates inhibition of the BRC4-RAD51 interaction by the T1526A peptide. RAD51, bound to a BRC4 peptide in the solid phase, was detected using a rabbit polyclonal antibody against RAD51. Disruption of this interaction, using soluble BRC peptides (BRC4, BRC4-T1526A and BRC5), caused a reduction in the colorimetric change induced by the action of an HRP-conjugated anti-rabbit secondary antibody on the substrate 3, 3’, 5, 5’- tetramethylbenzidine.
