# Supplementary material for: Interrogation of the Protein-Protein Interactions between Human BRCA2 BRC Repeats and RAD51 Reveals Atomistic Determinants of Affinity
Source: PLoS Comput Biol. 2011 Jul 14;7(7):e1002096. doi: 10.1371/journal.pcbi.1002096 (PMC3136434; doi:10.1371/journal.pcbi.1002096)
Supplement: Figure S8 — Sequence alignment of the BRC repeats used in FP assays. Sequence alignment of the eight human BRC repeats and a BRC4T08A mutation used in our FP assays, generated with ClustalW. The symbols on the bottom row denote the degree of conservation observed in each column: ‘*’ denotes that the residues in that column are identical in all sequences in the alignment, ‘:’ denotes that conserved substitutions have been observed and ‘.’ denotes that semi-conserved substitutions are observed. (PDF) [file pcbi.1002096.s008.pdf]

```
BRC1            -----NHSFGG-SFRTASNKEIKLSEHNIKKSKMFFKDIEEQY 37
BRC2            ---NEVGFRFG--FYSAHGTKLNVSTEALKAVK-LFSDIEN-- 35
BRC3            EQNIKDFETSDTFFQTASGKNISVAKESFNKIVNFFDKQPEEL 43
BRC4            ----KEPTLLG--FHTASGKKVKIAKESLDKVKNLFDEKEQ-- 35
BRC5            ----IENSALA--FYTSCSRKTSVSQTSLLEAKKWLREGIF-- 35
BRC6            --------VGPPAFRIASGKIVCVSHETIKKVKDIFTDS---- 31
BRC7            --------TCG-IFSTASGKSVQVSDASLQNARQVFSEIED-- 32
BRC8            -------AFSG--FSTASGKQVSILESSLHKVKGVLEEFDLI- 33
BRC4T08A        ----KEPTLLG--FHAASGKKVKIAKESLDKVKNLFDEKEQ-- 35
                             *  : .    :    :      : .
```

Figure S8. Sequence alignment of the eight human BRC repeats and a BRC4T08A mutation used in our FP assays, generated with ClustalW. The symbols on the bottom row denote the degree of conservation observed in each column: `*' denotes that the residues in that column are identical in all sequences in the alignment, `:' denotes that conserved substitutions have been observed and `.' denotes that semi-conserved substitutions are observed.
