# Supplementary material for: Interrogation of the Protein-Protein Interactions between Human BRCA2 BRC Repeats and RAD51 Reveals Atomistic Determinants of Affinity
Source: PLoS Comput Biol. 2011 Jul 14;7(7):e1002096. doi: 10.1371/journal.pcbi.1002096 (PMC3136434; doi:10.1371/journal.pcbi.1002096)
Supplement: Figure S9 — Reproducibility of alanine scanning for the RAD51-BRC6A interaction. Two computational alanine scans of the RAD51-BRC6A interface (grey), showing the reproducibility of the residues that contribute most to binding in long simulations. (PDF) [file pcbi.1002096.s009.pdf]

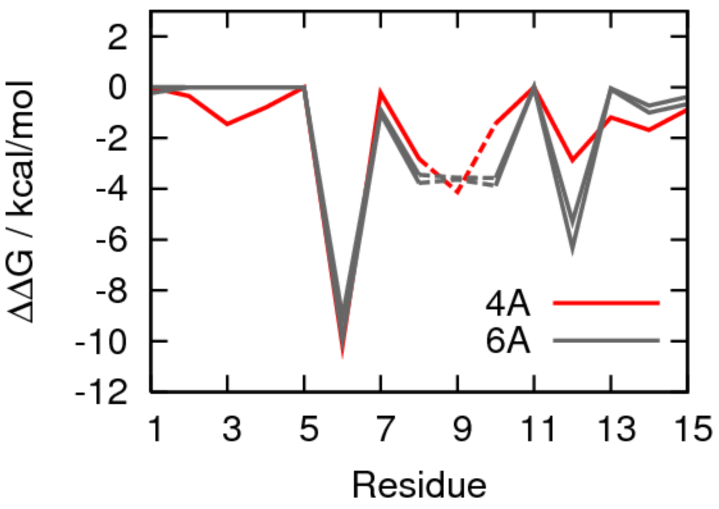

Figure S9. Two computational alanine scans of the RAD51-BRC6A interface (grey), showing the reproducibility of the residues that contribute most to binding in long simulations.
