# Supplementary material for: Interrogation of the Protein-Protein Interactions between Human BRCA2 BRC Repeats and RAD51 Reveals Atomistic Determinants of Affinity
Source: PLoS Comput Biol. 2011 Jul 14;7(7):e1002096. doi: 10.1371/journal.pcbi.1002096 (PMC3136434; doi:10.1371/journal.pcbi.1002096)
Supplement: Figure S10 — Glycine scan of the RAD51-BRC4A interface. Comparison between computational glycine scan and computational alanine scan of the RAD51-BRC4A interface. The differences between the two methods are small but may be significant around the hotspot region. (PDF) [file pcbi.1002096.s010.pdf]

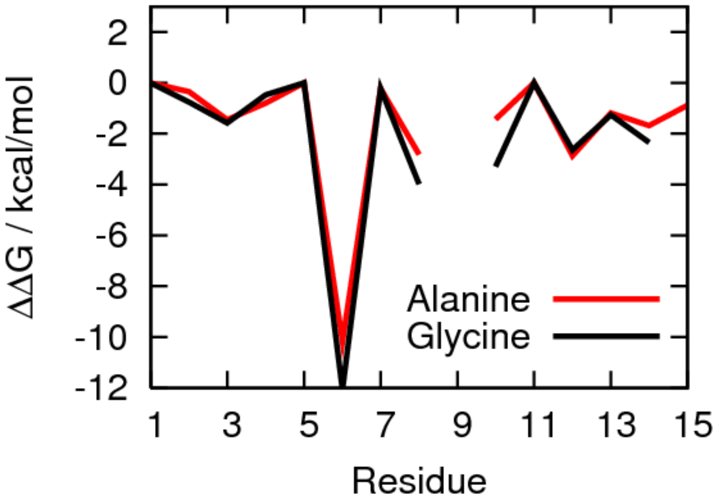

Figure S10. Comparison between computational glycine scan and computational alanine scan of the RAD51-BRC4A interface. The differences between the two methods are small but may be significant around the hotspot region.
