# Supplementary material for: Interrogation of the Protein-Protein Interactions between Human BRCA2 BRC Repeats and RAD51 Reveals Atomistic Determinants of Affinity
Source: PLoS Comput Biol. 2011 Jul 14;7(7):e1002096. doi: 10.1371/journal.pcbi.1002096 (PMC3136434; doi:10.1371/journal.pcbi.1002096)
Supplement: Figure S11 — Convergence of thermodynamic integration results. Convergence of the free energy of the T08A mutation in the RAD51-BRC4A complex using thermodynamic integration in three stages. Stage 1: Removal of partial charges from T08, stage 2: transformation of vdW parameters of T08 to A08 using soft-core potentials, stage 3: introduction of partial charges to A08. . T08 does not form any direct hydrogen bonds with RAD51, yet the majority of the free energy change is due to the removal of charge in stage 1. (PDF) [file pcbi.1002096.s011.pdf]

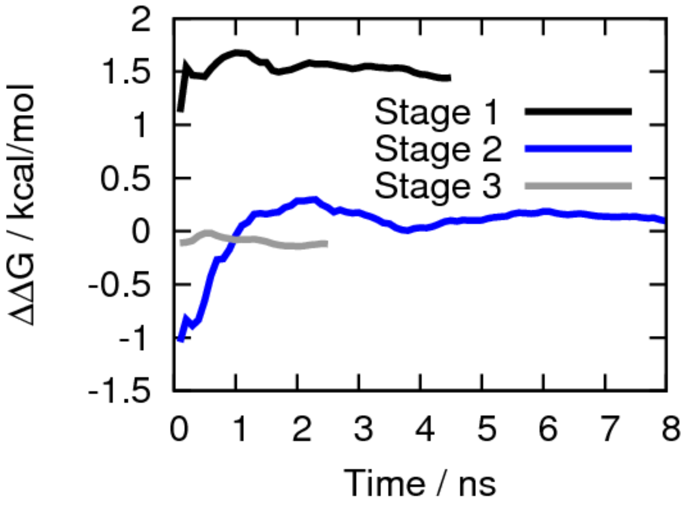

ΔG / kcal/mol	Stage 1	Stage 2	Stage 3
Protein	31.6	1.0	2.7
Water	30.2	0.9	2.8

Figure S11. Convergence of the free energy of the T08A mutation in the RAD51-BRC4A complex using thermodynamic integration in three stages. Stage 1: Removal of partial charges from T08, stage 2: transformation of vdW parameters of T08 to A08 using soft-core potentials, stage 3: introduction of partial charges to A08. ΔΔG = ΔG(protein) - ΔG(water). T08 does not form any direct hydrogen bonds with RAD51, yet the majority of the free energy change is due to the removal of charge in stage 1.
