# Supplementary material for: Interrogation of the Protein-Protein Interactions between Human BRCA2 BRC Repeats and RAD51 Reveals Atomistic Determinants of Affinity
Source: PLoS Comput Biol. 2011 Jul 14;7(7):e1002096. doi: 10.1371/journal.pcbi.1002096 (PMC3136434; doi:10.1371/journal.pcbi.1002096)
Supplement: Text S1 — Additional MM-PBSA simulations of cancer-associated BRC mutants. (PDF) [file pcbi.1002096.s012.pdf]

Supporting Text S1

Interrogation of the Protein-Protein Interactions between Human BRCA2 BRC Repeats and RAD51 Reveals Atomistic Determinants of Affinity

Daniel J. Cole,[1] Eeson Rajendra,[2] Meredith Roberts-Thomson,[3] Bryn Hardwick,[3] Grahame J. McKenzie,[3] Mike C. Payne,[1] Ashok R. Venkitaraman,[2,3] Chris-Kriton Skylaris[4]

[1] Theory of Condensed Matter Group, Cavendish Laboratory, University of Cambridge, J. J. Thomson Avenue, Cambridge CB3 0HE, UK

[2] MRC Cancer Cell Unit, Hutchison/MRC Research Centre, Hills Road, Cambridge CB2 0XZ, UK

[3] Cambridge Molecular Therapeutics Programme, Hutchison/MRC Research Centre, Hills Road, Cambridge CB2 0XZ, UK

[4] School of Chemistry, University of Southampton, Highfield, Southampton SO17 1BJ, UK

Additional MM-PBSA simulations of cancer-associated BRC mutants

In addition to the mutations discussed in the main text (BRC2A S08P, BRC4A T08A and BRC7A T08I), we have also performed MM-PBSA calculations on BRC repeats containing single residue mutations that increase the net positive charge of the ligand, which we would expect to increase the free energy of binding with the negatively charged RAD51 receptor.

Figure S5(a) reveals that both BRC1A T08R and BRC4A G11R mutations do indeed result in a net gain in binding free energy. However, both results come with caveats. The alanine scan (Figure S5(c)) of the G11R mutation shows negligible contribution to binding from the R11 side chain and so the position, as well as the magnitude of the charge, is clearly a relevant factor. The small gain in binding free energy appears to instead be a result of limitations in the MM-PBSA method in describing the mobile N- and C-termini of the peptide, which supports our use of computational alanine scanning to discern each residue's contribution to binding.

The BRC1A T08R mutation affects the critical hotspot region and, therefore, alters

the observed binding modes. The alanine scan reveals that the binding contributions are very similar to the RAD51-BRC1A interface except at the site of the mutation. R08 binds to D187 (in a similar fashion to S08 in BRC2A), replacing K12 and enhancing the occupancy of the S10-D187 hydrogen bond (55% in wildtype to 89% in the mutant), which is stronger than in wildtype BRC1A. Although it is thought that mutations disrupt BRC repeat binding to RAD51, the T08R mutation may in fact be detrimental as it binds too strongly to RAD51 – a functional consequence of uncoupling precise modes of RAD51 regulation engendered by the collocation of two distinct modules within each repeat binding in modes antagonistic or permissive to RAD51 oligomerisation.
